# Hf/Zr Superlattice-Based High‑κ Gate Dielectrics with Dipole Layer Engineering for Advanced CMOS

**DOI:** 10.1021/acsnano.5c15062

**Published:** 2026-01-08

**Authors:** Taeyoung Song, Sanghyun Kang, Yu-Hsin Kuo, Jiayi Chen, Lance Fernandes, Nashrah Afroze, Mengkun Tian, Hyoung Won Baac, Changhwan Shin, Asif Islam Khan

**Affiliations:** † School of Electrical and Computer Engineering, 1438 Georgia Institute of Technology, Atlanta, Georgia 30332, United States; ‡ Department of Electrical and Computer Engineering, 35017 Sungkyunkwan University, Suwon 16419, Republic of Korea; § School of Electrical Engineering, 34973 Korea University, Seoul 02841, Republic of Korea

**Keywords:** ultrathin high-κ, superlattice, dipole engineering, reliability, RMG process, GAAFET

## Abstract

Advanced logic transistors require gate dielectrics that achieve subnanometer equivalent oxide thickness (EOT), suppress leakage, and satisfy three key requirements: (i) compatibility with RMG-like high-temperature processing, (ii) sufficient *V*_th_ tunability for multi-*V*_th_ design, and (iii) high device reliability. However, meeting all of these requirements at once has been difficult with conventional high-κ systems. In this work, we demonstrate that our Hf/Zr-based gate stacks quantitatively satisfy these conditions. (i) After a 700 °C N_2_ anneal, the HZH superlattice achieves EOT = 7.3 Å, lower than conventional HfO_2_-only stacks (8.5 Å) while maintaining comparable leakage. (ii) Embedding a 3 Å Al_2_O_3_ dipole within the HfO_2_/ZrO_2_/HfO_2_ superlattice (HZHA) breaks the conventional dipole trade-off, achieving an 8.4 Å EOT—lower than the 9.0 Å of a standard HfO_2_/Al_2_O_3_ stack—while providing a > 200 mV *V*_FB_ shift, thereby enabling multi-*V*_th_ tuning without compromising scaling. (iii) Furthermore, under −2 V negative-bias temperature stress at 125 °C for 100 s, HZHA and HA exhibit comparable *V*_FB_ drifts of 87 mV and 97 mV, respectively, confirming that strong *V*_th_ tunability and subnanometer EOT can be achieved without compromising stability. In addition to these quantitative advances, this study reveals previously unreported physical insights into the dipole behavior and interfacial diffusion in ultrathin Hf/Zr multilayers. These results establish HZHA as an RMG-compatible, *V*_th_-tunable, low-EOT dielectric platform capable of supporting logic scaling beyond the 1 nm frontier.

## Graphical abstract

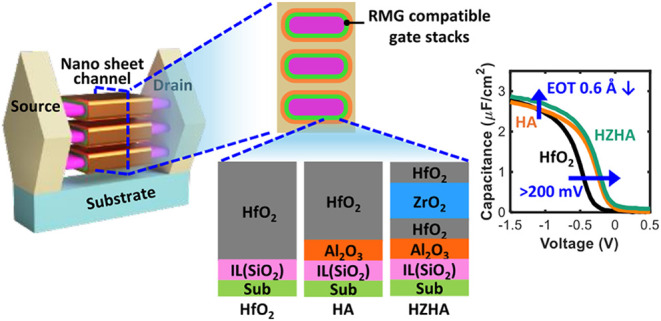

## Introduction

The demand for more powerful and energy-efficient computing, driven by applications in artificial intelligence (AI), high-performance computing, and edge devices, has accelerated the miniaturization of logic transistors. In accordance with Moore’s Law, continued scaling to smaller device dimensions improves computational efficiency and integration density but also presents new challenges in device design and materials integration. Gate-all-around (GAA) field-effect transistors (FETs) have emerged as a key solution to maintain electrostatic control as transistor width is reduced.^1–6^ Compared with traditional FinFETs, GAA transistors offer superior electrostatic control and reduced short-channel effects, making them well-suited for continued scaling (Figure 1A). By completely wrapping the gate around the channel, GAA FETs suppress short-channel effects and improve performance over traditional FinFETs, enabling further scaling of CMOS technology. However, the relentless drive toward nanometer-scale dimensions imposes severe constraints on integrating critical materials—such as work function metals and gate dielectrics—into an ever-shrinking volume (Figure 1B). In particular, gate dielectrics must provide extremely high capacitance in an ultrathin layer while also allowing tunable threshold voltages (*V*_th_) to meet the performance requirements. Stronger gate electrostatic control is needed to mitigate short-channel effects, and higher gate capacitance is required to sustain drive current for high-speed operation.^7–12^

**Figure 1:**
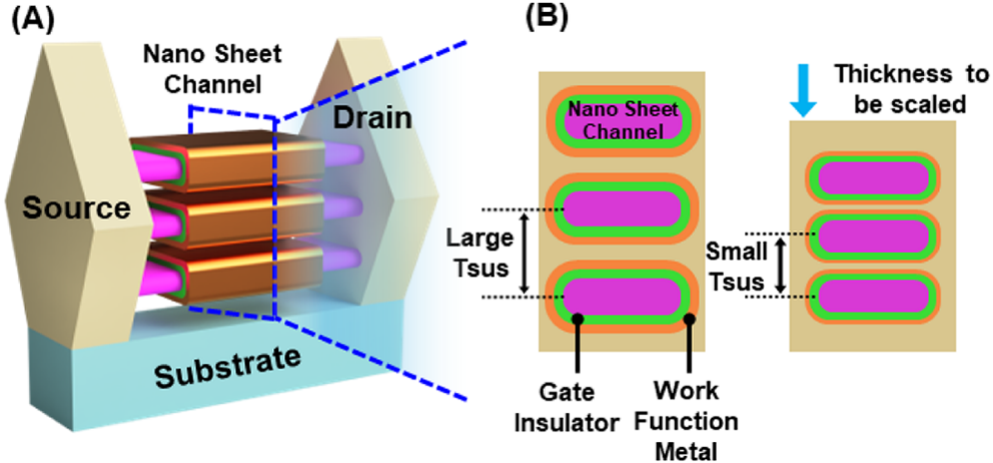
Need for ultrathin gate oxides with high-κ dielectrics and low gate leakage. (A) 3D schematic of a GAAFET structure. (B) Cross-sectional view of stacked nanosheets in a GAAFET, highlighting the increasingly constrained space for gate insulators and work function metals in advanced logic transistors.

One approach to achieve high capacitance at a reduced thickness is the integration of advanced high-κ dielectric materials. Recent studies have explored laminated and superlattice oxides based on HfO_2_, ZrO_2_, and Hf–Zr mixed oxides (HZO), particularly near the morphotropic phase boundary (MPB), to enable further EOT scaling.^13–19^ While many of these efforts have focused on metal–insulator–metal capacitors for ferroelectric memory or DRAM applications and sometimes metal–oxide–semiconductor (MOS) structures, high-temperature annealing steps are often omitted, raising concerns about thermal stability. In practical CMOS integration, gate dielectrics must withstand subsequent high-temperature processes, such as dopant activation or defect annealing, without degradation, to ensure long-term device reliability. Postdeposition annealing (PDA) is critical for passivating defects and improving material properties; inadequate thermal treatment can leave the dielectric vulnerable to instability under the operating conditions. As such, understanding the impact of high-temperature annealing on advanced high-κ gate stacks is essential for their integration into future technology nodes.^20,21^

Another key challenge in aggressively scaled EOT is the increase in gate leakage current due to direct tunneling, which can lead to excessive power consumption. An intrinsic trade-off exists between a dielectric’s permittivity (κ) and its bandgap. High-κ materials enable a reduction in the gate oxide thickness, enhancing electrostatic gate control. However, their relatively low bandgap energies can lead to increased leakage currents under operating conditions.^22,23^ Therefore, optimizing the κ-bandgap balance is critical to minimizing leakage while maintaining gate capacitance, particularly in the subnanometer EOT regime.

Moreover, as physical dimensions shrink, incorporating work function metals to modulate the threshold voltage (*V*_th_) becomes increasingly difficult due to the limited gate volume. In this context, dipole engineering within the gate stack provides a “volume-less” method for *V*_th_ tuning. By introducing ultrathin dielectric interlayers with differing oxygen areal densities (typically at the high-κ/SiO_2_ interface), dipoles, which shift the flatband voltage (*V*_FB_) without adding physical thickness, can be formed. Various dipole materials, such as La_2_O_3_ and Al_2_O_3_, and their thickness dependence have been extensively studied.^24–26^ However, systematic investigations into the optimal position of the dipole layer within the gate stack remain limited. Strategic placement of dipole layers could provide additional degrees of freedom for precise *V*_th_ control in highly scaled devices.

In this study, we systematically investigate four gate dielectric stacks—HfO_2_-only, HfO_2_/ZrO_2_/HfO_2_ (HZH), ZrO_2_/HfO_2_/ZrO_2_ (ZHZ), and HZO/ZrO_2_/HZO (HZZ)—to evaluate their impact on EOT and gate leakage current. A high-temperature 700 °C PDA is found to markedly improve flatband voltage stability under negative bias stress (NBS), with the HZH and ZHZ configurations demonstrating significantly enhanced reliability compared to the conventional HfO_2_-only baseline. Furthermore, we systematically explored dipole engineering by incorporating an ultrathin Al_2_O_3_ layer at various positions within the stack to modulate the threshold voltage. While such Al_2_O_3_ integration inevitably increases the EOT due to the low dielectric constant of Al_2_O_3_, this drawback is mitigated by employing the HZH structure. Consequently, the optimized HZHA gate stack (HZH with an Al_2_O_3_ dipole) achieves a favorable combination of reduced EOT, maintaining *V*_th_ tunability, high-temperature process compatibility, and enhanced reliability, positioning it as a strong candidate for future CMOS logic devices. These findings highlight the HZHA gate stack as a promising platform for enabling EOT scaling, threshold voltage control, and long-term reliability in advanced CMOS logic technologies.

## Results and Discussion

### Investigation of High-κ Gate Stack Structures for Enhanced Capacitance, Leakage Control, and Reliability

We first compared the electrical characteristics of three gate stack structures—HfO_2_-only, HZH, and ZHZ—each with the same total thickness but different layer compositions. The MOS capacitors were fabricated following the process flow shown in Figure S1, with a total gate insulator thickness of 21 Å and additional details explained in the TEM and EDS images (Figures S2 and S3). Figure 2A illustrates the layer configurations for the HfO_2_ reference and the laminated HZH and ZHZ stacks with layer thicknesses in angstroms. Capacitance–voltage (*C*–*V*) and gate leakage current density–voltage (*J*_g_–*V*) measurements were performed to evaluate the performance of these configurations (Figure 2B–G).

**Figure 2:**
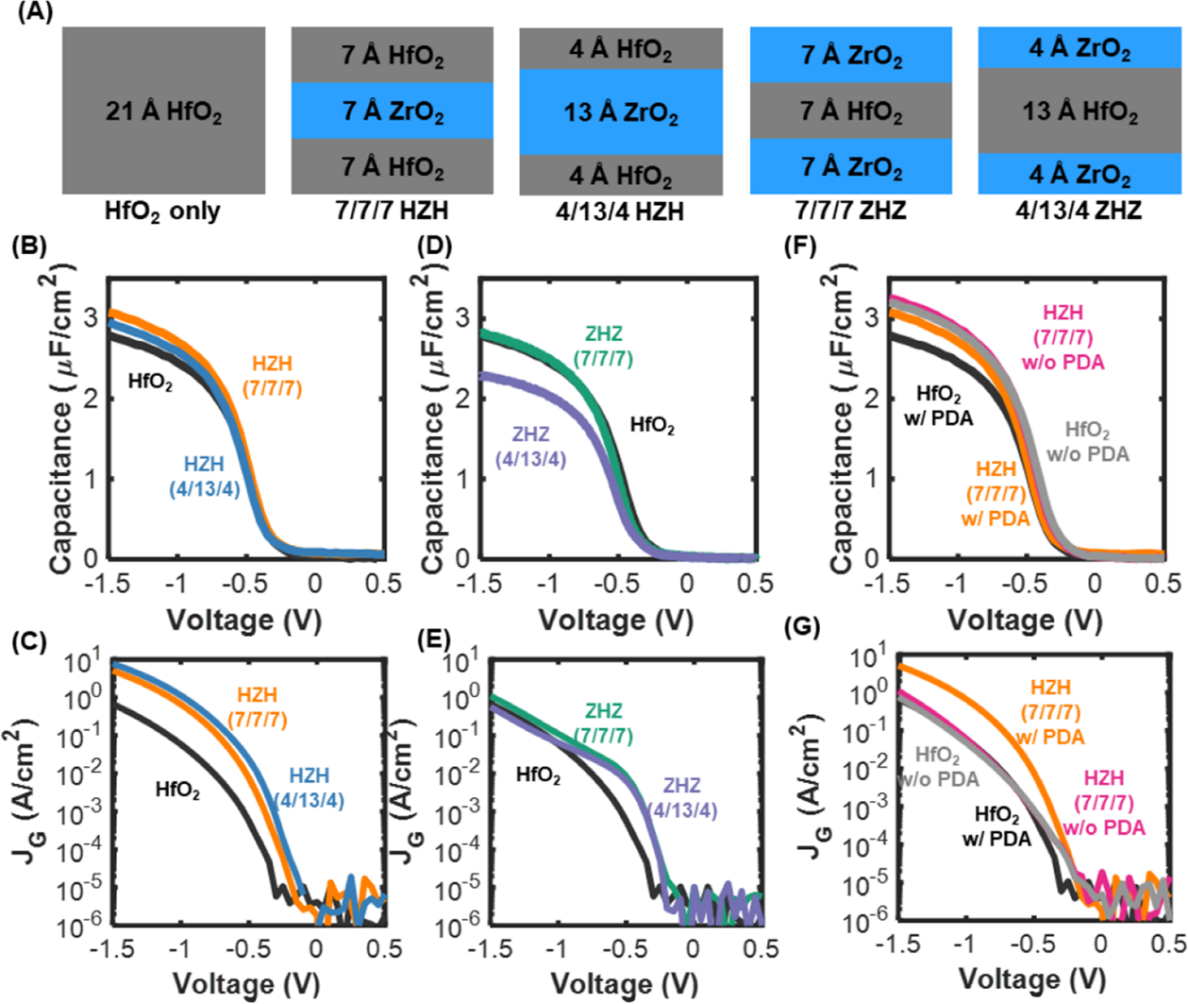
Gate stack engineering for enhanced capacitance and minimized gate leakage current. (A) Schematic of the HfO_2_-only, HZH, and ZHZ gate stack configurations with varying thicknesses. (B) and (C) Capacitance–voltage (*C*–*V*) and gate leakage current–voltage (*J*_g_–*V*) characteristics of HfO_2_ and HZH gate stacks. (D) and (E) Capacitance–voltage (*C*–*V*) and gate leakage current–voltage (*J*_g_–*V*) characteristics of HfO_2_ and ZHZ gate stacks. (F) and (G) Capacitance–voltage (*C*–*V*) and gate leakage current–voltage (*J*_g_–*V*) characteristics of HfO_2_ and HZH stacks, both with and without postdeposition annealing (PDA).

This demonstrates that the HZH stack consistently exhibits a higher capacitance than the HfO_2_-only sample, indicating an increased effective dielectric constant due to the incorporation of the intermediate ZrO_2_ layer. In contrast, the ZHZ stack shows a capacitance that is comparable to or slightly lower than that of HfO_2_-only, underscoring the strong influence of layer ordering on the overall permittivity. Notably, the symmetric 7/7/7 Å HZH laminate achieved an EOT of 7.3 Å, a significant improvement over the 8.5 Å EOT of the HfO_2_-only control. A thicker asymmetric HZH stack (4/13/4 Å) yielded a slightly higher EOT of 7.8 Å, confirming that the HfO_2_:ZrO_2_ thickness ratio impacts the dielectric performance. A similar trend is observed in the ZHZ configuration: the symmetric 7/7/7 Å ZHZ stack attained an EOT of 8.4 Å, whereas the less optimized 4/13/4 Å ZHZ stack showed a much larger EOT of 10.6 Å. These results highlight the importance of both the stacking sequence and individual layer thickness optimization in achieving the desired high-κ performance.

Significant research has been devoted to achieving a low EOT in HfO_2_- and ZrO_2_-based dielectrics through compositional and structural engineering. Recent studies on ultrathin ferroelectric HfO_2_–ZrO_2_ superlattice capacitors have shown that a coexistence of the ferroelectric orthorhombic (o) phase and the antiferroelectric tetragonal (t) phase can effectively reduce the EOT, leveraging negative capacitance or high permittivity effects at the phase boundary.^13,16,18^ In our HZH laminate, X-ray diffraction (Figure S4) confirms the presence of mixed crystal phases: a dominant o-(111) peak at 2θ ≈ 30.4 ° (accounting for 78.13% of the film) alongside a weaker t-(011) peak (21.25%) and a minor monoclinic phase residual. This phase coexistence is a key factor contributing to the reduced EOT of the HZH stack compared with a single-layer HfO_2_ film. The ZrO_2_ incorporation helps stabilize the desirable orthorhombic phase of HfO_2_, which has higher permittivity, thereby boosting the capacitance for a given thickness.

Besides phase stabilization, the HfO_2_:ZrO_2_ thickness ratio is pivotal in optimizing the dielectric performance. Our comparison of 7/7/7 Å versus 4/13/4 Å HZH stacks shows that the distribution of Hf and Zr dictates the o versus t phase balance and, in turn, the attainable EOT. Sufficient HfO_2_ content is required to stabilize the orthorhombic phase; when the ZrO_2_ fraction becomes excessive—as in the 4/13/4 Å stack—the structure favors the tetragonal phase, leading to a thicker effective oxide. Precise control of individual layer thicknesses is therefore essential to reproducibly target the desired phase composition and permittivity.

The gate leakage characteristics of the stacks are shown in Figure 2C, E. The HZH stack exhibits only a slight increase in leakage current compared to the HfO_2_-only device, while the ZHZ stack maintains a leakage level similar to that of the HfO_2_ reference. This suggests that inserting ZrO_2_ layers can enhance capacitance without dramatically compromising the leakage current. Leakage current densities in both HZH and ZHZ laminates remain comparable to or only slightly higher than those of pure HfO_2_ across the operating range, consistent with their band edge alignment: HfO_2_’s larger bandgap (∼5.8 eV) provides the dominant barrier, while the modest introduction of ZrO_2_ (∼5.4 eV) only marginally increases tunneling probability, leaving overall leakage acceptable for logic applications.^22,23^ However, careful tuning of the ZrO_2_ layer’s thickness and position is required to balance the trade-off between higher κ and potential increases in leakage. The HZH stack shows a favorable trade-off: it achieves a substantially lower EOT (higher capacitance) than the HfO_2_-only baseline, with only a minor leakage penalty, making it attractive for future high-performance devices. The ZHZ (7/7/7) stack, on the other hand, offers moderate capacitance (similar to HfO_2_) but with stable leakage behavior, illustrating how different laminate designs can target either maximum capacitance or minimized leakage according to application needs.

To assess the influence of high-temperature processing, we subjected each gate stack to a 700 °C PDA for 30 s in N_2_ and compared the results with those of unannealed controls. Without PDA, the HfO_2_ only stack exhibited an exceptionally low EOT of 7.1 Å and low gate leakage. Following the 700 °C anneal, its EOT increased to 8.5 Å, and the leakage current rose by roughly 50% (Figure 2F, G). The HZH stack displayed a more modest EOT shift—from 7.0 Å to 7.3 Å—but experienced a proportionally larger rise in leakage current.

A notable observation in our study is that both EOT and gate leakage current density (*J*_g_) increased after high-temperature annealing, even for a conventional HfO_2_ sample. As the 700 °C 30-s annealing process is not a high heat budget process enough to transit the phase of crystalline HfO_2_, the primary cause of this degradation is the formation of interfacial silicates at the SiO_2_/high-κ interface during annealing. At elevated temperatures, HfO_2_ or ZrO_2_ in contact with the interfacial SiO_2_ layer can react to form hafnium silicate (HfSiO_4_) or zirconium silicate (ZrSiO_4_). While these silicate phases are thermally stable, they possess a dielectric constant significantly lower than that of pure HfO_2_ or ZrO_2_, resulting in an effective thickness increase. Indeed, silicate formation is a well-known limiting factor for EOT scaling in high-κ dielectrics.^27^ Meanwhile, prior to PDA, the as-deposited EOT values exhibited a similar value to the best value of HfO_2_–ZrO_2_ systems from the previously reported paper.^13^ However, after PDA, the HZH sample would have another effect, which is the intermixing of HZH and interaction within the interface between HfO_2_ and ZrO_2_ which leads to more increase in the gate leakage.

In our stacks, the ZrO_2_-containing samples were slightly more susceptible to silicate-induced EOT increases than the HfO_2_-only sample. This is expected because ZrSiO_4_ formation occurs at a lower temperature than HfSiO_4_.^27^ Thus, in the ZHZ stack, the bottom ZrO_2_ layer can readily convert to ZrSiO_4_ at 700 °C, significantly reducing the effective κ at the critical interface. In HZH, the bottom interface is HfO_2_, which is less prone to silicate formation at that temperature; therefore, the EOT penalty is less severe. Consequently, the detrimental impact of silicate formation is more pronounced in the ZHZ structure compared to that in HZH. Another factor contributing to increased leakage after annealing is grain growth in the polycrystalline HfO_2_ and ZrO_2_ layers. Prolonged or high-temperature anneals can enlarge crystal grains and possibly form grain boundaries that serve as leakage pathways, slightly raising *J*_g_.^28,29^

We next evaluated the NBS reliability of the gate stacks by measuring the flatband voltage shift under prolonged negative gate bias. MOS capacitors were stressed at −2 V for durations from 1 to 2000 s, and the *C*–*V* curves were periodically measured to extract *V*_FB_ (Figure 3A–D). The detailed NBS measurement pulse is illustrated in Figure S6. Tests were done at both room temperature (RT) and elevated temperatures of 85 and 125 °C to assess thermal acceleration of instability. The unannealed HfO_2_ sample showed a pronounced negative *V*_FB_ shift of −182 mV after just 100 s of stress at RT, indicating substantial charge trapping in the dielectric (primarily hole trapping and interface trap generation).^30,31^ In contrast, the HfO_2_ sample that received the 700 °C PDA exhibited a much smaller shift (−53 mV at 100 s and −73 mV at 2000 s), highlighting that high-temperature annealing dramatically improves bias stability by reducing preexisting defects.^32,33^ This underscores the necessity of PDA in mitigating charge trapping and ensuring long-term reliability of high-κ gate stacks.^32,33^

**Figure 3:**
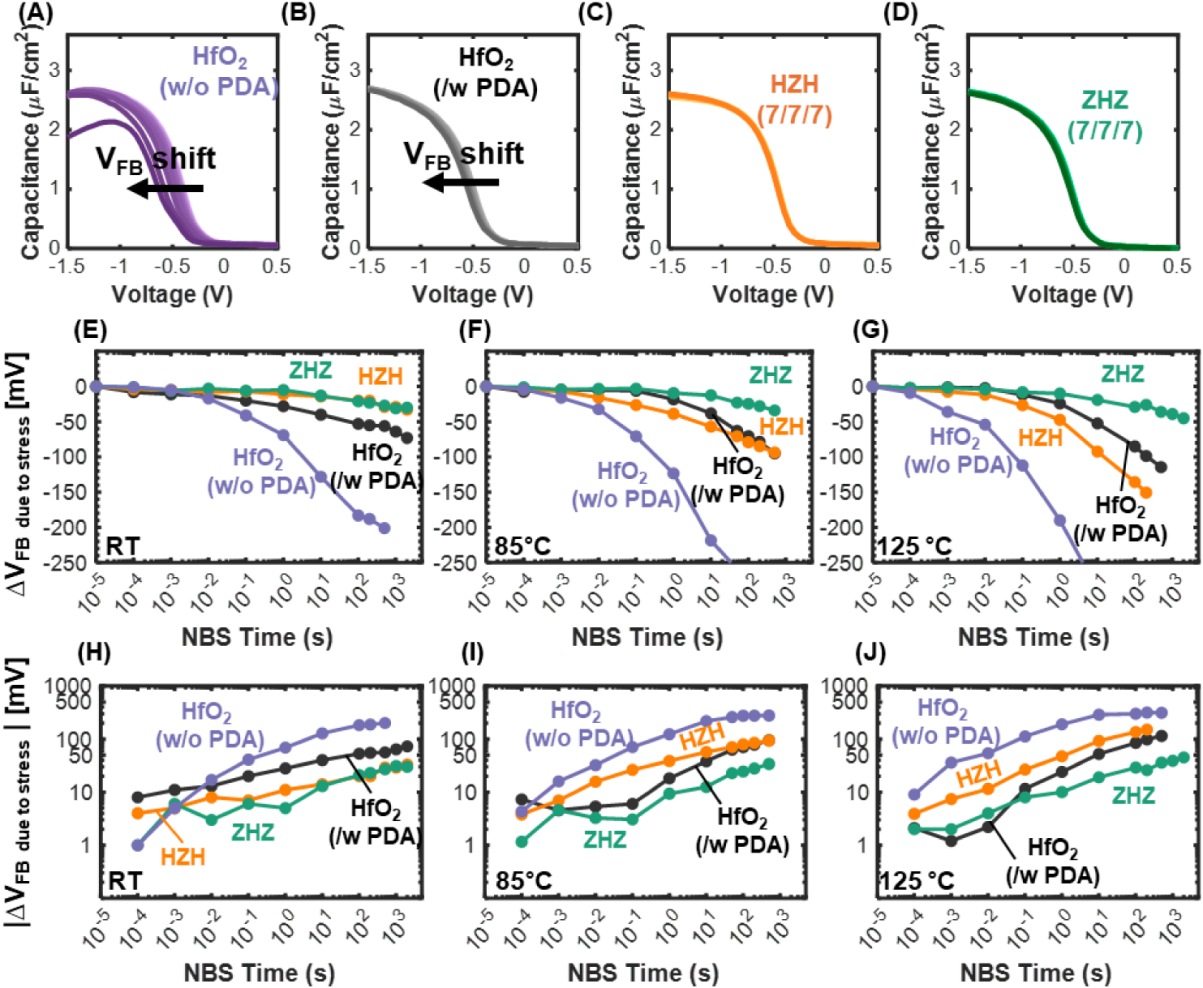
NBS measurement for evaluating the reliability of various gate stacks. (A)–(D) Capacitance–voltage (*C*–*V*) measurements under a negative bias (−2 V) over a duration of 0 to 2000 s. (E)–(G) Flatband voltage shift due to negative bias stress on a linear scale measured at (E) room temperature (RT), (F) 85 °C, and (G) 125 °C. (H)–(J) present the same data on a logarithmic scale at (H) RT, (I) 85 °C, and (J) 125 °C.

The NBS characteristics further highlight the temperature-dependent reliability differences among the gate stacks. At room temperature, the HZH stack exhibits better NBS stability than the annealed HfO_2_ control, while its performance becomes comparable at 85 °C and slightly worse at 125 °C. In contrast, the ZHZ stack maintains consistently superior reliability across all measured temperatures. At RT, the HZH and ZHZ samples exhibit only −33 and −30 mV shifts, respectively, after 2000 s of stress. At 125 °C, after 100 s of −2 V stress, annealed HfO_2_ shows a −85 mV shift; on the other hand, the HZH and ZHZ stacks exhibit −135 mV and −29 mV shifts, respectively. Even after 2000 s at 125 °C, the ZHZ stack maintained the smallest degradation (−45 mV). These trends, summarized in Figure 3E–G, indicate that although PDA-treated samples exhibit broadly comparable stability at RT, the ZHZ stack clearly outperforms the others under high-temperature stress. Figure 3H–J presents the same data on a logarithmic *y*-scale. The approximately linear behavior in the log–log plots confirms a power-law dependence of Δ*V*_FB_ on stress time, consistent with charge-trapping and interface-state generation mechanisms. The activation energies extracted from the Arrhenius analysis of Δ*V*_FB_ at 100 s (Figure S8) reveal a distinct asymmetry: HZH exhibits a substantially higher *E*_a_, whereas both annealed HfO_2_ and the ZHZ superlattice show shallow activation energies. This divergence indicates asymmetric Hf↔Zr intermixing during high-temperature annealing—pronounced in HZH but strongly suppressed in ZHZ, where the ZrO_2_ outer layers act as diffusion barriers. The deeper trap states formed through intermixing in HZH account for its stronger temperature acceleration, whereas the limited intermixing in ZHZ yields shallow trap kinetics and superior high-temperature reliability.

The superior robustness of ZHZ can be understood by examining its initial defect landscape and interfacial chemistry. Silicates such as HfSiO_4_ and ZrSiO_4_ are known to exhibit lower defect densities than their respective binary oxides.^34,35^ Because ZrSiO_4_ forms at a lower temperature than HfSiO_4_,^35–37^ the ZHZ structure is more likely to develop a ZrSiO_4_-rich transition layer during PDA. This interpretation is strongly supported by the *D*_it_ analysis in Figure S7: ZrO_2_-containing stacks exhibit significantly lower *D*_it_ after PDA than their HfO_2_ counterparts, particularly in the high-Δ*E* region. Thus, the ZHZ stack begins with a lower density of preexisting interface traps, reducing the number of sites available for immediate occupation under a negative bias. This lower initial trap density plays a decisive role in the early onset NBS response. With fewer traps available for rapid filling, the ZHZ stack shows much smaller Δ*V*_FB_ shifts during both short and long stress durations. The stress-bias dependence in Figure S9 further supports this conclusion: although Δ*V*_FB_ increases with both stress voltage and time, as expected for trap generation under higher fields, the ZHZ stack consistently exhibits the smallest Δ*V*_FB_ across all bias conditions. Overall, the combination of a lower initial trap density, favorable ZrSiO_4_ interfacial formation, and a balanced superlattice arrangement results in the ZHZ gate stack demonstrating the strongest NBS reliability across all temperatures and stress conditions.

In summary, each gate stack configuration offers distinct advantages. The HZH structure provides enhanced capacitance compared to HfO_2_ alone, making it appealing for applications that demand high drive current and performance. The ZHZ structure, while not improving the EOT beyond the HfO_2_ baseline, delivers superior reliability under stress, which is critical for applications requiring long-term stability. These findings underscore the importance of structural engineering in high-κ gate stacks to balance capacitance and leakage performance against reliability.

### Impact of Drive-In Temperature on Different Dipole Layer Locations

To systematically study dipole layer placement, we fabricated devices with a 3 Å Al_2_O_3_ interfacial layer inserted at three different locations in an HfO_2_-based gate stack: at the bottom interface (adjacent to the SiO_2_ interfacial layer), in the middle of HfO_2_, or at the top of HfO_2_ (just below the metal gate). Figure 4A illustrates these configurations. Each of these samples was subjected to annealing at 700 °C, 800 °C, and 900 °C to examine how thermal budget influences dipole formation and activation. The flatband voltage shift (Δ*V*_FB_) due to the dipole was extracted from the *C*–*V* curves for each case (Figure 4B–D).

**Figure 4:**
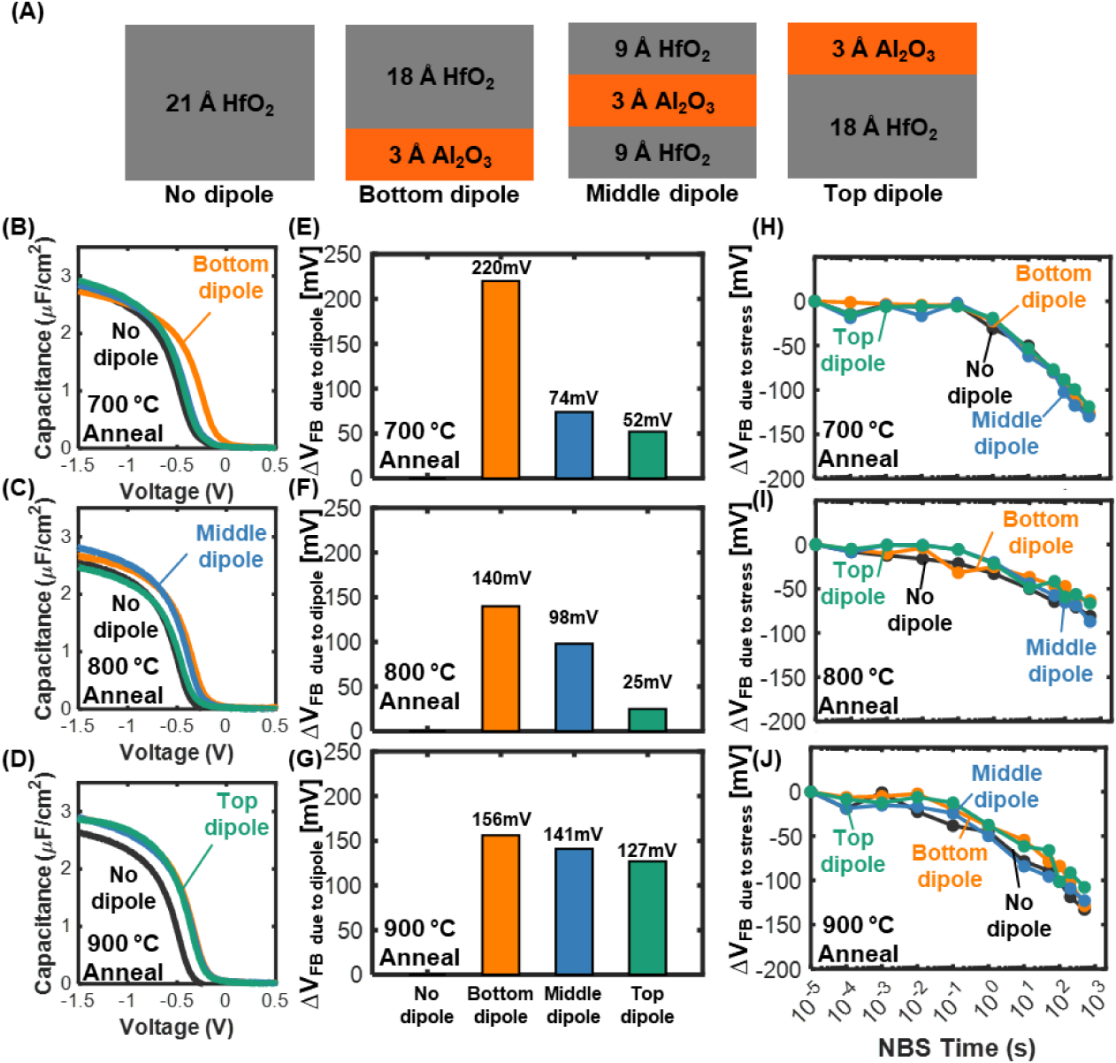
Dipole engineering study with different Al_2_O_3_ locations and annealing temperatures. (A) Schematic of gate stack configurations with different dipole locations. (B)–(D) Capacitance–voltage (*C*–*V*) measurements of samples annealed at 700 °C, 800 °C, and 900 °C, respectively. (E)–(G) Flatband voltage shift of samples annealed at 700 °C, 800 °C, and 900 °C, respectively. (H)–(J) NBS measurement results at elevated temperature (125 °C) for samples annealed at 700 °C, 800 °C, and 900 °C, respectively.

At 700 °C, a substantial *V*_FB_ shift of +220 mV was observed only for the sample with the dipole at the bottom interface, whereas the midlayer and top-layer dipole samples showed much smaller shifts of +74 mV and +52 mV, respectively (Figure 4E). This behavior indicates that at this relatively lower annealing temperature, the dipole is fully activated only when positioned at the bottom interface, likely because it is directly adjacent to the SiO_2_ interface, where interfacial dipole formation is most favorable. The corresponding *C*–*V* curves (Figure 4B) confirm the large *V*_FB_ shift for the bottom-dipole device and much smaller shifts for the other two.

The mechanism of dipole formation at oxide interfaces is rooted in differences in the oxygen areal density between two adjoining layers. Generally, when a layer with a higher oxygen concentration is adjacent to one with a lower concentration, oxygen ions tend to diffuse into the lower concentration region upon annealing. This leaves behind oxygen vacancies in the higher concentration material. These oxygen vacancies (positively charged) at the interface align with oxygen anions (negatively charged) in the neighboring layer, thereby forming an electric dipole at the interface. The direction of the dipole depends on the direction of oxygen diffusion.^38^

In our experiments, we employed Al_2_O_3_ as the dipole-forming layer. When Al_2_O_3_ is placed at the bottom of the gate stack (directly on the SiO_2_ interfacial layer), oxygen from Al_2_O_3_ diffuses into SiO_2_ during annealing, creating vacancies in Al_2_O_3_. This produces dipoles oriented from the SiO_2_ side toward the Al_2_O_3_, which induces a positive *V*_FB_ shift.^39^ By contrast, if the Al_2_O_3_ layer is located in the middle or top of the HfO_2_ stack, then it is initially separated from SiO_2_ by HfO_2_. Upon annealing, some Al and O interdiffusion can occur through HfO_2_, and dipoles can still form at the nearest SiO_2_ interface. However, the farther the Al_2_O_3_ is from the SiO_2_, the less oxygen exchange occurs, resulting in a much smaller dipole and hence a smaller *V*_FB_ shift. This explains why, at 700 °C, only the bottom dipole showed a significant effect, whereas the middle and top dipoles did not.

When the annealing temperature increased to 800 °C, the dipole in the middle of the stack became more activated, showing a larger Δ*V*_FB_ of 98 mV, while the bottom dipole’s effect slightly decreased to 140 mV (Figure 4F). At 900 °C, the top dipole configuration exhibited a pronounced *V*_FB_ shift (127 mV), nearly catching up to Δ*V*_FB_ of 156 mV with the bottom dipole structure, which remained roughly constant between 800 °C and 900 °C, as shown in Figure 4G. These trends suggest that higher annealing temperatures enhance the diffusion of oxygen and subsequent dipole formation, even for dipoles that are initially farther from the SiO_2_ interface. In other words, a sufficiently high-temperature “drive-in” can activate dipole formation throughout the high-κ stack, not just at the bottom interface. To activate dipoles located away from the immediate interface, we used a higher-temperature drive-in anneal (up to 900 °C). At such high temperatures, the diffusion lengths of oxygen in HfO_2_ increase substantially, enabling oxygen from Al_2_O_3_ even in the middle or upper part of the stack, to reach the SiO_2_ eventually, thereby forming measurable dipoles (Figure 4G).^24,40^

Conversely, for the bottom dipole, as the net dipole effect at the bottom interface slightly diminishes beyond 700 °C, there appears to be an optimal annealing window. An intriguing behavior was observed for the bottom dipole case: the *V*_FB_ shift caused by the bottom Al_2_O_3_ layer was largest at 700 °C and then reduced at 800 °C and 900 °C. This nonmonotonic trend can be understood by considering the so-called mirror-plane effect in high-κ dielectrics. In a HfO_2_-based dielectric stack, there can be a self-regulating interdiffusion of cations and anions at interfaces that tends to neutralize dipoles.^25^ At moderate temperatures, enough oxygen diffuses to form a strong dipole at the SiO_2_/Al_2_O_3_ interface, while Hf and other cation diffusion is limited. At much higher temperatures, however, oxygen diffusion becomes extremely pronounced and can extend beyond the first interface: in our case, oxygen from Al_2_O_3_ can diffuse not only into SiO_2_ but also upward into the adjacent HfO_2_ layer. Concurrently, Hf cations can diffuse into the Al_2_O_3_ region, to some extent. This upward anion/cation intermixing generates an opposite interfacial dipole component—i.e., a counter-dipole—that partially cancels the intended dipole at the SiO_2_ interface. Specifically, since HfO_2_ has a lower oxygen areal density than Al_2_O_3_, oxygen will also diffuse from Al_2_O_3_ into HfO_2_ at high temperature, creating vacancies in Al_2_O_3_ near the HfO_2_ side. This induces an opposing dipole oriented from Al_2_O_3_ toward HfO_2_.^38,41^ At 900 °C, this counter-dipole effect remains present but is not strictly monotonic: the net *V*_FB_ shift is still lower than at 700 °C but slightly higher than at 800 °C, consistent with temperature-dependent competition between dipole formation and counter-dipole cancellation. In summary, these results demonstrate that excessive high-temperature diffusion introduces a counter-dipole that reduces the net dipole strength, and careful control of the annealing temperature is required to maximize the beneficial dipole at the SiO_2_ interface while avoiding excessive counter-dipole formation due to overdiffusion.

We further evaluated the dipole-engineered stacks under negative bias stress (NBS) across multiple temperatures, including room temperature (RT), 85 °C, and 125 °C. The elevated-temperature results at 125 °C are presented in Figure 4H–J, while the corresponding RT and 85 °C data are provided in Figure S10. The NBS pulse conditions were consistent with those described in Figure S6. As expected, applying −2 V stress induced a negative shift in *V*_FB_ in all cases due to charge trapping. Importantly, however, the magnitude of the *V*_FB_ shift after stress was similar for devices with bottom, middle, or top dipoles and largely independent of whether they were annealed at 700, 800, or 900 °C. In other words, within the measurement resolution, dipole layer placement and the drive-in anneal temperature did not significantly affect the NBS-induced degradation. All devices showed only modest *V*_FB_ shifts under prolonged stress, indicating that the introduction of the Al_2_O_3_ dipole layer does not compromise the reliability of the gate stack.

The impact of dipole insertion on the channel mobility and reliability is an important consideration for practical devices. Some studies have reported that if a dipole-forming layer such as Al_2_O_3_ is directly interfaced with SiO_2_, it can lead to mobility degradation in MOSFETs, possibly due to scattering from the dipole interface.^24^ Conversely, other reports suggest that dipoles can improve reliability by providing a source of excess oxygen to passivate interface traps, thereby reducing interface state density (*D*_it_).^42^ Our reliability tests on capacitors with various dipole placements (Figure 4H–J) showed no adverse effect of the dipole on NBS-induced *V*_FB_ shifts, even at elevated temperatures (125 °C); all configurations were essentially equally stable, and in fact, the absolute shifts were very small. This suggests that, at least in terms of bias stress, adding an Al_2_O_3_ dipole layer does not degrade the dielectric reliability. It will be necessary, however, to conduct bias-temperature instability (BTI) tests on actual transistors to fully evaluate any subtle effects dipoles might have on channel mobility or interface trap generation over operating lifetimes.

To evaluate threshold voltage tunability, we investigated the dependence of electrical characteristics on Al_2_O_3_ thickness in the gate stack structures, as illustrated in Figure 5A. Three different Al_2_O_3_ thicknesses—1 Å, 3 Å, and 5 Å—were incorporated by partially replacing the 21 Å HfO_2_ layer, ensuring that the total dielectric thickness remained constant across all samples. This approach allowed for a controlled assessment of dipole strength while minimizing capacitance variations caused by total thickness changes. The capacitance–voltage (*C*–*V*) measurements (Figure 5B) show a clear positive shift of the *C*–*V* curve as the Al_2_O_3_ thickness increases, accompanied by a decrease in maximum capacitance. The extracted *V*_FB_ values exhibit a strong correlation with Al_2_O_3_ thickness (Figure 5C): +99 mV for 1 Å, +169 mV for 3 Å, and +229 mV for 5 Å, confirming that the dipole effect intensifies with increasing Al_2_O_3_ thickness. This enhancement is attributed to interfacial dipole formation, driven by differences in the oxygen areal density between adjacent dielectric layers. Thicker Al_2_O_3_ layers promote greater oxygen diffusion, resulting in a stronger dipole moment and a larger positive shift in *V*_FB_. The EOT values, extracted from the *C*–*V* curves (Figure 5C), show a clear degradation trend with increasing Al_2_O_3_ thickness due to its intrinsically lower dielectric constant compared to that of HfO_2_. Specifically, EOT increased by 0.35 Å, 0.97 Å, and 1.43 Å for 1 Å, 3 Å, and 5 Å of Al_2_O_3_, respectively. These results clearly demonstrate the intrinsic EOT–*V*_FB_ trade-off in conventional HfO_2_/Al_2_O_3_ dipole stacks, motivating the use of intrinsically low-EOT platforms—such as the Hf/Zr superlattice—to enable meaningful *V*_th_ modulation without compromising scaling. In this context, it is important to evaluate how dipole-based *V*_th_ modulation integrates with low-EOT superlattice structures such as HZH and to determine the implications for advanced CMOS design.

**Figure 5:**
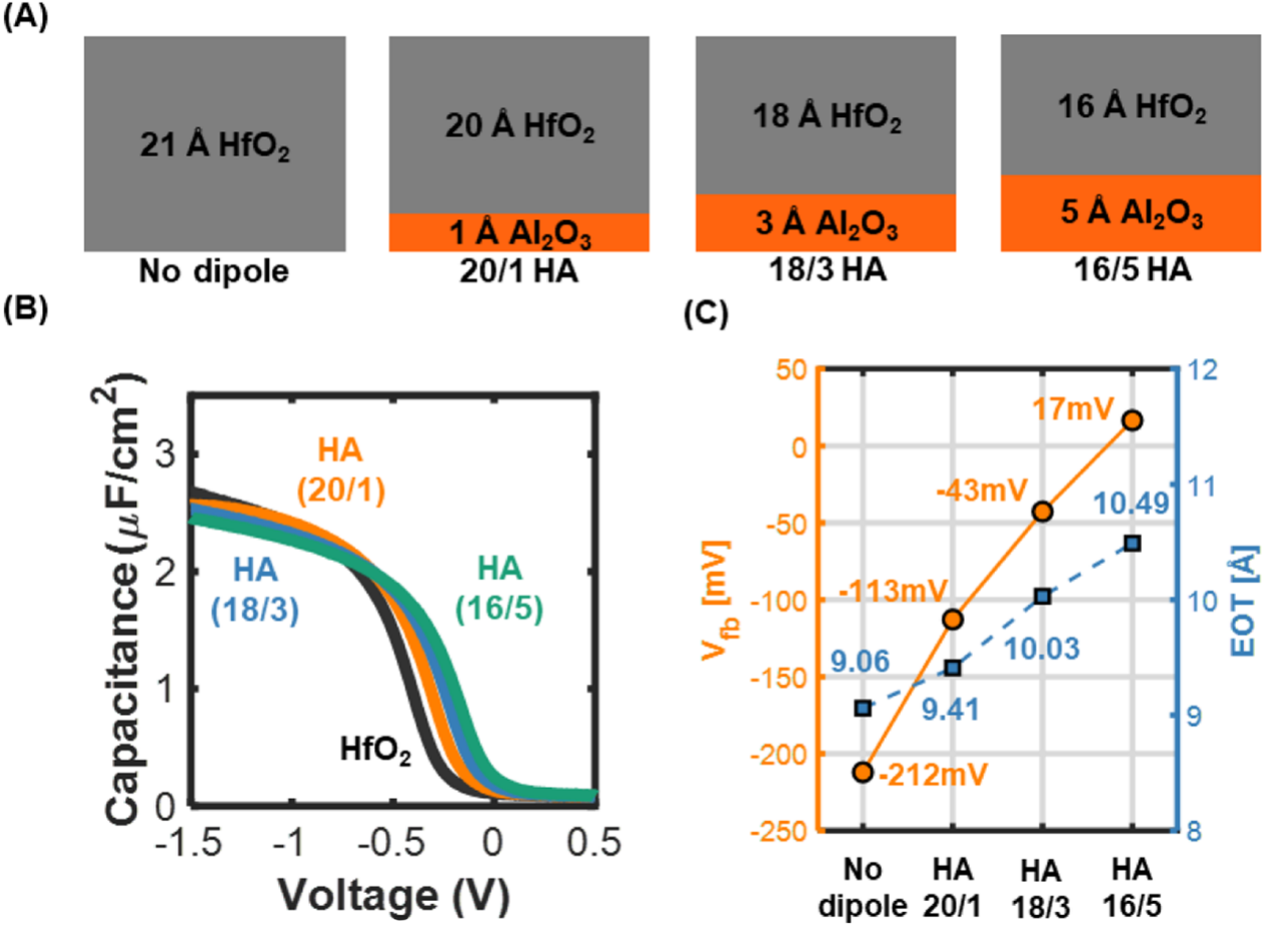
Demonstration of the EOT–*V*_FB_ shift trade-off by modulating Al_2_O_3_ dipole thickness in HfO_2_/Al_2_O_3_ gate stacks. (A) Schematic of gate stack configurations with different dipole thicknesses. (B) Capacitance–voltage (*C*–*V*) measurements of HA stacks with varying Al_2_O_3_ thicknesses. (C) Flatband voltage (*V*_FB_) and equivalent oxide thickness (EOT) values as a function of Al_2_O_3_ dipole thickness.

### *V*_FB_ Modulation in Superlattice High-κ Gate Stacks through Dipole Engineering

To investigate the effect of incorporating an Al_2_O_3_ dipole into previously examined gate stack structures, we embedded the dipole layer into HfO_2_/ZrO_2_ superlattice configurations. By doing so, we leverage the enhanced permittivity of the laminate while gaining *V*_FB_ modulation of the dipole, thus balancing electrostatic control with capacitance retention. We extended our investigation beyond the HfO_2_-only dielectric to three laminated high-κ stacks (HZH, ZHZ, and HZZ), each incorporating a bottom-interface Al_2_O_3_ dipole (Figure 6A).

**Figure 6:**
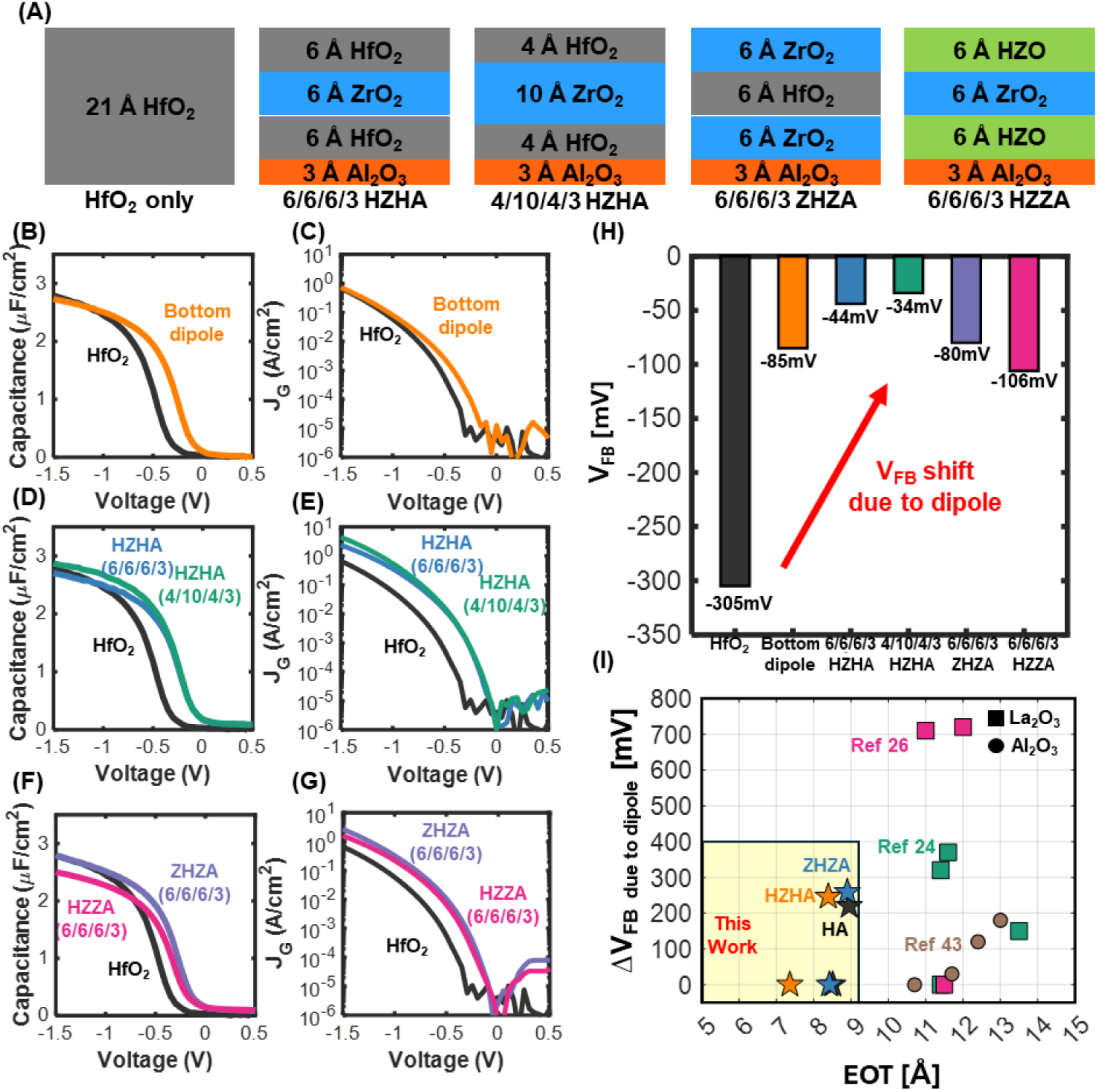
Incorporation of a dipole into the Hf/Zr superlattice structure for *V*_th_ tunability and enhanced high-κ performance. (A) Schematic of the HfO_2_-only, HZH, ZHZ, and HZZ gate stack configurations with the dipole layer positioned at the bottom. (B) and (C) Capacitance–voltage (*C*–*V*) and gate leakage current–voltage (*J*_g_–*V*) characteristics for HfO_2_ and HfO_2_ with a bottom dipole. (D) and (E) Capacitance–voltage (*C*–*V*) and gate leakage current–voltage (*J*_g_–*V*) characteristics for HfO_2_ and HZH gate stacks with a bottom dipole. (F) and (G) Capacitance–voltage (*C*–*V*) and gate leakage current–voltage (*J*_g_–*V*) characteristics for HfO_2_ and ZHZ/HZZ gate stacks with a bottom dipole. (H) Flatband voltage of different gate stacks. Regardless of the gate stack configuration, those incorporating a bottom dipole exhibit a flatband voltage shift. (I) Benchmark comparison of the studied gate stacks with results from other studies.

Figure 6B–G presents *C*–*V* and *J*_g_–*V* data for several representative cases with a bottom dipole. The introduction of the 3 Å Al_2_O_3_ layer at the HfO_2_/SiO_2_ interface consistently induces a positive flatband voltage shift, confirming the formation of the intended dipole. Quantitatively, adding the dipole to a pure HfO_2_ gate stack (sample “HA,” referring to HfO_2_ + bottom dipole Al_2_O_3_) shifted *V*_FB_ by about +220 mV relative to the undoped HfO_2_, but also increased the EOT from 8.5 Å to 9.0 Å. Importantly, the gate leakage current density remained essentially unchanged at the dipole (Figure 6B, C). This demonstrates that the Al_2_O_3_ interlayer can modulate *V*_th_ without incurring a severe leakage penalty, although a slight capacitance reduction is observed.

The stack composition with the dipole significantly influences the overall device behavior. Among the high-κ laminates tested, the HZH stack showed the lowest EOT even with the dipole in place—even slightly lower than that of conventional HfO_2_ without a dipole. In contrast, the ZHZ stack with a dipole exhibited a lower capacitance and higher leakage compared to its no-dipole counterpart, underlining that the base HfO_2_/ZrO_2_ configuration can matter as much as the dipole itself. We further examined two HZH stack variants with different HfO_2_/ZrO_2_ thickness ratios to see how they interact with the dipole. Without a dipole, the symmetric 7/7/7 HZH had a lower EOT than that of the asymmetric 4/13/4 HZH, as discussed earlier. When the dipole was added at the bottom interface, an interesting result emerged: the 4/10/4/3 HZHA stack achieved a lower EOT (8.4 Å) than the 6/6/6/3 HZHA stack (9.2 Å). In other words, the laminate with a higher ZrO_2_ proportion benefitted more from the dipole integration in terms of maintaining a low EOT. By contrast, other dipole-integrated configurations like ZHZA (ZrO_2_/HfO_2_/ZrO_2_ + Al_2_O_3_) and HZZA (HZO/ZrO_2_/HZO + Al_2_O_3_) exhibited significantly higher EOTs (8.9 Å and 10.0 Å, respectively) despite the dipole, due to their less favorable base structures. These findings emphasize that to fully exploit dipole engineering, one must jointly optimize the high-κ stack geometry. A well-tuned HfO_2_:ZrO_2_ ratio can offset the intrinsic EOT cost of the Al_2_O_3_ layer, thereby maximizing the capacitance while still enabling *V*_FB_ shifts.

We calculated the flatband voltages for all of the different gate stacks to compare the effect of the dipole (Figure 6H). In each case, incorporation of the Al_2_O_3_ dipole resulted in a discernible *V*_FB_ shift relative to the corresponding structure without a dipole. The magnitude of the shift varied depending on the stack composition (with HZH showing the largest shift and HZZ the smallest), but the qualitative presence of a shift in HZH, ZHZ, and HZZ confirms successful dipole formation in all these high-κ matrices. This demonstrates the versatility of dipole engineering across various dielectric heterostructures; even when the baseline *V*_FB_ values of HZH, ZHZ, and pure HfO_2_ devices are nearly identical, adding the dipole reliably moves *V*_FB_ in the positive direction.

Figure 6I provides a performance benchmarking of our dipole-integrated stacks in terms of flatband voltage shift (Δ*V*_FB_) versus EOT. As noted, the HfO_2_-only + dipole stack (HA) incurs a slight EOT increase compared to pure HfO_2_. In contrast, the HZH + dipole stack manages to achieve a lower EOT than pure HfO_2_ due to the ZrO_2_ layers while still obtaining a sizable *V*_FB_ shift from the dipole. This underlines the promise of combining laminate high-κ dielectrics with dipole layers: the laminate addresses the capacitance requirement, and the dipole provides *V*_th_ tuning with manageable trade-offs.

Figure 6I benchmarks the flatband voltage tunability (Δ*V*_FB_) against equivalent oxide thickness for various dipole-engineered gate stacks reported in the literature. Prior studies employing La_2_O_3_ or Al_2_O_3_ interlayers have demonstrated substantial Δ*V*_FB_ (>300–700 mV) but at the cost of large EOTs (20–50 Å). In contrast, our HfO_2_–ZrO_2_ superlattice platform enables the integration of a 3 Å Al_2_O_3_ dipole within sub-10 Å EOT stacks: the HZHA and ZHZA structures both exhibit Δ*V*_FB_ shifts in excess of 200 mV while maintaining EOTs of 8.4 and 9.0 Å, respectively. This performance region (highlighted in yellow) lies well below the EOT regime of previous dipole-only approaches, illustrating that coupling dipole engineering with high-κ superlattice dielectric stacks is a highly effective strategy to achieve aggressive scaling and threshold-voltage tunability simultaneously.

We further evaluated the NBS reliability of these dipole-integrated superlattice stacks. Figure 7A, B compares the NBS response of four stacks—HfO_2_, HA, HZH, and HZHA—at room temperature and 125 °C. At both temperatures, introducing an ultrathin Al_2_O_3_ dipole directly on the SiO_2_ interlayer (HA) does not degrade reliability; the Δ*V*_FB_ versus time trace remains similar to, or modestly better than, that of the conventional HfO_2_. This behavior suggests that the dipole interface supplies excess oxygen during stress, promoting the partial conversion of HfO_2_ to Hf–Al silicate and thereby passivating vacancy sites that would otherwise trap holes. When the dipole is embedded within the HfO_2_/ZrO_2_/HfO_2_ superlattice (HZHA), the stack retains the superlattice’s intrinsic NBS robustness at room temperature and, importantly, sustains comparable or slightly smaller Δ*V*_FB_ than conventional HfO_2_ at 125 °C. The combination of Zr-mediated trap suppression and Al-assisted interfacial silicate formation therefore enables the HZHA architecture to deliver threshold voltage tunability without sacrificing high-temperature reliability, affirming its suitability for advanced CMOS logic applications. Figure 7C, D plots the 100 s negative-bias stress Δ*V*_FB_ against EOT for all investigated stacks at room temperature and 125 °C, respectively. The shaded “design goal” region denotes the quadrant combining EOT < 8.5 Å with |*V*_FB_| < 100 mV. At both temperatures, the HZHA stack lies within this target region, exhibiting an EOT of 8.4 Å alongside minimal Δ*V*_FB_ (<20 mV at RT and <100 mV at 125 °C). For comparison, the HA stack provides tunability but falls outside the design window because of its larger EOT, whereas the conventional HfO_2_ stack without PDA exhibits a substantial flatband voltage shift. Notably, HZHA maintains low Δ*V*_FB_ under high-temperature stress—demonstrating that embedding the dipole within the HfO_2_–ZrO_2_ superlattice uniquely satisfies the competing requirements of subnanometer scaling and robust negative-bias reliability. In parallel, our NBS data highlight the strong potential of the ZHZ architecture for applications requiring enhanced reliability under both thermal and electrical stress. By naturally forming a more inert interface and maintaining a high overall κ, the ZHZ stack achieves an excellent balance of performance and stability. It points to a broader strategy where interface chemistry is engineered for reliability in tandem with bulk high-κ improvements.

**Figure 7:**
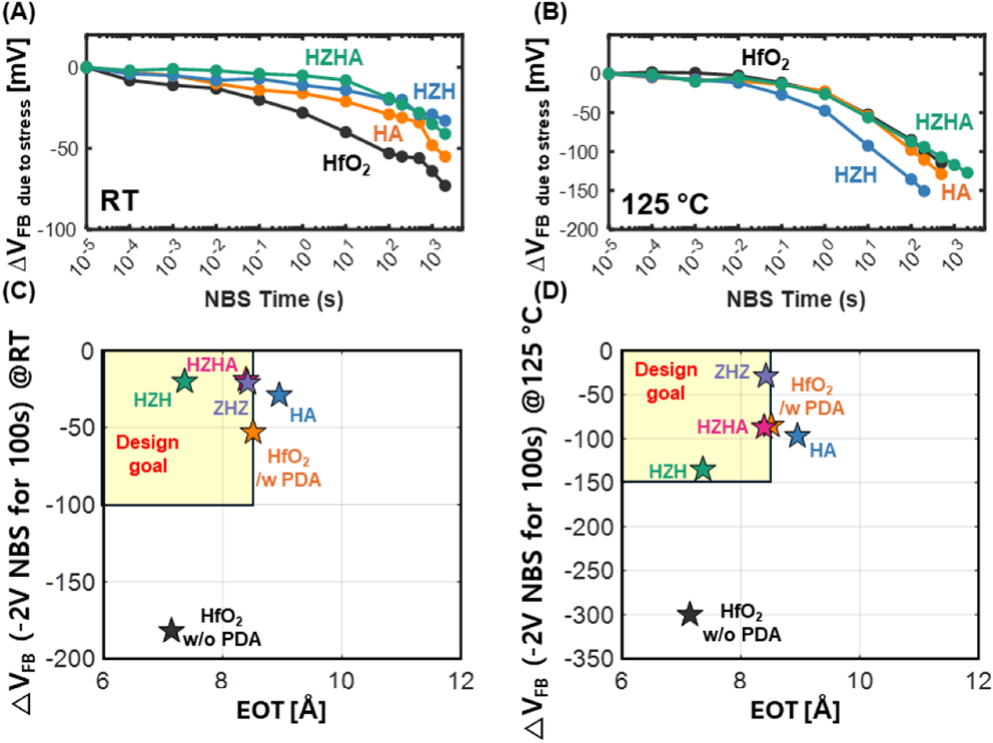
NBS measurements of gate stacks with incorporated dipole layer. (A) and (B) Flatband voltage shift (Δ*V*_FB_) as a function of negative bias stress (NBS) time measured at room temperature and 125 °C for various gate stack configurations. (C) and (D) Flatband voltage shift (*V*_FB_) versus EOT plot for the structures investigated in this work.

Finally, by incorporating an Al_2_O_3_ dipole into our best-performing high-κ laminates (HZH, ZHZ, HZZ), we demonstrated clear flatband voltage modulation in each case (Figure 6H). The fact that the baseline *V*_FB_ of HZH, ZHZ, and HZZ without dipole could be made nearly identical through an appropriate metal gate work function, and then each could be shifted positively by adding the dipole, confirms the viability of dipole engineering across different high-κ systems. The dipole did incur a minor EOT due to a lower dielectric constant of Al_2_O_3_ than that of HfO_2_ and ZrO_2_,^22,23^ but the leakage current remained largely unchanged. Notably, the HZHA (4/10/4/3) stack achieved a slightly lower EOT than even some no-dipole configurations, illustrating that a well-optimized HfO_2_/ZrO_2_ composition can compensate for the Al_2_O_3_’s lower κ. This implies that by tuning the Hf:Zr ratio in concert with dipole integration, one can simultaneously maximize the dipole effect and minimize the EOT penalty, ultimately preserving or even enhancing the overall capacitance.

Table 1 synthesizes the key performance metrics of the investigated gate stacks—EOT, gate leakage density, flatband voltage shifts after negative bias stress at both room temperature and 125 °C, *V*_th_ tunability, and compatibility with replacement metal gate (RMG) processing. Relative to the HfO_2_ control, HZH superlattices deliver thinner EOTs without sacrificing significant leakage integrity, and the 700 °C postdeposition anneal substantially suppresses NBS-induced *V*_FB_ drift. Incorporating an ultrathin Al_2_O_3_ dipole layer increases the EOT for any stack; however, the high-κ HZH framework offsets this penalty, such that the dipole-integrated HZHA stack retains competitive EOT while gaining intrinsic *V*_th_ adjustability. Moreover, HZHA maintains NBS stability at elevated temperature and endures the high-temperature anneal, confirming full compatibility with advanced RMG flows. Collectively, the data position HZHA as the most balanced candidate for future logic nodes, combining aggressive scaling, threshold voltage control, and robust reliability.

**Table 1:**
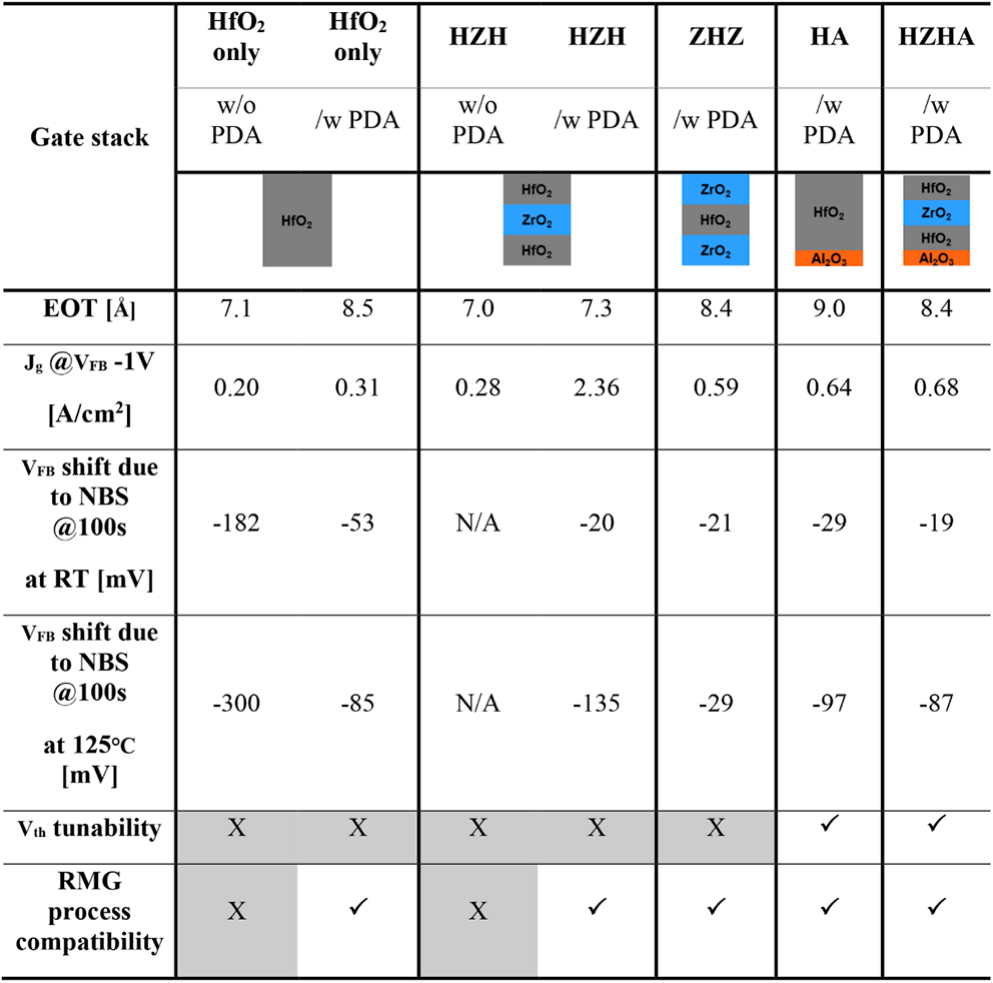
Summary Table of Characteristics for Various Types of Gate Stacks

Table 2 benchmarks MFIS gate-stack performance reported in prior studies, comparing annealing conditions, EOT/CET, and *V*_th_ tunability. The color coding indicates CMOS process compatibility, with green representing the most favorable conditions, yellow indicating intermediate performance, and red indicating the least favorable outcome. Notably, the gate dielectric developed in this work withstands high-temperature annealing up to 700 °C while still achieving a low EOT enabled by the HfO_2_/ZrO_2_ superlattice structure. Furthermore, the incorporation of an ultrathin Al_2_O_3_ dipole layer within the superlattice allows controllable *V*_FB_ tuning, demonstrating that both aggressive EOT scaling and threshold-voltage engineering can be simultaneously realized within a CMOS-compatible thermal budget.

**Table 2:**
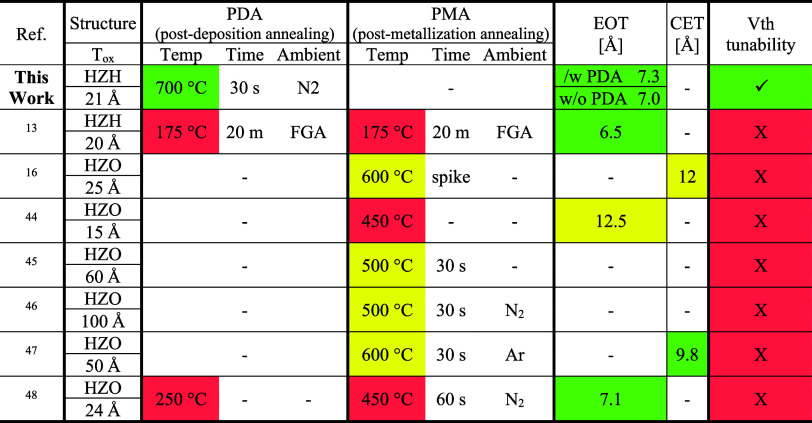
Benchmark Table of MFIS Gate-Stack Performance against State-of-the-Art Works, Comparing Annealing Conditions, EOT/CET, and *V*_th_ Tunability^a^ ^43,44,45,46,47,48^

a Color coding qualitatively benchmarks CMOS compatibility: green is the most favorable, yellow is intermediate, and red is the least favorable.

## Conclusions

In this work, we demonstrate that HfO_2_/ZrO_2_ superlattice gate stacks incorporating ultrathin Al_2_O_3_ dipole layers simultaneously achieve aggressive EOT scaling, low leakage, a tunable flatband voltage, and high-temperature CMOS compatibility. The optimized HZH laminate delivers a 7.3 Å EOT—well below the 8.5 Å of conventional HfO_2_—while dipole integration enables >200 mV *V*_FB_ modulation with an 8.4 Å EOT in the HZHA stack. Although ZHZ does not further reduce EOT, it exhibits outstanding negative-bias stress resilience, reducing the 125 °C Δ*V*_FB_ from 85 mV (HfO_2_) to 29 mV. In addition to these device-level advances, the study reveals two previously unreported physical insights—position-dependent dipole activation with a high-temperature counter-dipole response and asymmetric Hf↔Zr intermixing that dictates trap energetics—offering new understanding of dipole formation, ionic diffusion, and defect behavior in ultrathin high-κ heterostructures. Collectively, these results position dipole-engineered HfO_2_/ZrO_2_ superlattices as a promising pathway toward subnanometer, high-performance gate dielectrics for future CMOS nodes.

## Method

The detailed device fabrication process is described in Note S1, while the device measurement methods for *C*–*V*, *J*_g_–V, and NBS are explained in Note S2. Additionally, the parameter extraction method for *V*_FB_, EOT, and *J*_g_ at *V*_FB_ = 1 V is provided in Note S3.

## Supplementary Material



## Notes

21 Å gate-oxide MOSCAP (Figure S1); cross-section TEM images of multiple gate-stack architectures (Figure S2); EDS mapping of 21 Å HfO_2_ gate stack (Figure S3); XRD analysis of HfO_2_-ZrO_2_-HfO_2_ superlattice gate oxide (Figure S4); frequency-dependent capacitance–voltage characteristics of various gate stacks (Figure S5); NBS stress comparison of gate stacks under different stress biases (Figure S6); Δ*V*_FB_ as a function of stress time under negative bias stress of −2 V for four gate stack configurations (Figure S7); Arrhenius plot of Δ*V*_FB_ measured at a stress time of 100 s under a negative bias stress of −2 V (Figure S8); Δ*V*_FB_ measured at stress times of 1 ms, 10 ms, 100 ms, 1 s, and 10 s under negative bias stress ranging from 0 V to −3 V (Figure S9); Δ*V*_FB_ under NBS stress at RT and 85 °C for different dipole locations and annealing temperatures (Figure S10) (PDF)
