# Correction to “Hf/Zr Superlattice-Based High‑κ Gate Dielectrics with Dipole Layer Engineering for Advanced CMOS”

**DOI:** 10.1021/acsnano.6c01243

**Published:** 2026-02-16

**Authors:** Taeyoung Song, Sanghyun Kang, Yu-Hsin Kuo, Jiayi Chen, Lance Fernandes, Nashrah Afroze, Mengkun Tian, Hyoung Won Baac, Changhwan Shin, Asif Islam Khan

In the originally published article, the funding source information in the Acknowledgments section was incomplete. The Acknowledgments section has been corrected to include support from the National Science Foundation CAREER Award (Award No. 2047880). The corrected Acknowledgments section is provided below.

This work was supported by SRC GRC 2024 Logic and Memory Devices (#3235.001), the National Science Foundation CAREER Award (#2047880), and the National Research Foundation of Korea (NRF) grant funded by the Korea government (MSIT) (No. RS-2023-00260527). We also acknowledge Seunghoon Sung, Ashish Penumatcha, Uygar Avci, and Ashish Agrawal of Intel Foundry Technology Research (Hillsboro, Oregon, USA, and Ireland) for their valuable contributions and insightful discussions.

This correction does not affect the results, discussion, or conclusions of the original paper.

