# Supplementary material for: Hf/Zr Superlattice-Based High‑κ Gate Dielectrics with Dipole Layer Engineering for Advanced CMOS
Source: ACS Nano. 2026 Jan 8;20(2):2092–103. doi: 10.1021/acsnano.5c15062 (PMC12825384; doi:10.1021/acsnano.5c15062)
Supplement: Supplementary file 1 [file nn5c15062_si_001.pdf]

Supporting information

Hf/Zr Superlattice-Based High-κ Gate Dielectrics with Dipole Layer Engineering for Advanced CMOS

AUTHOR NAMES

Taeyoung Song,[1,4,*] Sanghyun Kang, [1,2,4,*], Yu-Hsin Kuo[1], Jiayi Chen[1], Lance Fernandes[1], Nashrah Afroze[1], Mengkun Tian[1], Hyoung Won Baac[2], Changhwan Shin[3] and Asif Islam Khan[1]

AUTHOR ADDRESS

[1]School of Electrical and Computer Engineering, Georgia Institute of Technology, Atlanta, GA, 30332, USA

[2]Department of Electrical and Computer Engineering, Sungkyunkwan University, Suwon, 16419, Republic of Korea

[3]School of Electrical Engineering, Korea University, Seoul, 02841, Republic of Korea

[4]These authors contributed equally

Table of Contents

Figure S1: 21 Å Gate-Oxide MOSCAP: Fabrication Steps, Stack Schematic, and Chip Layout

Figure S2: Cross-Section TEM Images of Multiple Gate-Stack Architectures

Figure S3: EDS Mapping of 21 Å HfO_2 Gate Stack

Figure S4: XRD analysis of HfO_2-ZrO_2-HfO_2 superlattice gate oxide

Figure S5. Frequency-dependent capacitance–voltage characteristics of various gate stacks

Figure S6. NBS stress comparison of gate stacks under different stress biases

Figure S7. Flatband voltage shift (ΔV_{FB}) as a function of stress time under negative bias stress of –2 V for four gate stack configurations: unannealed conventional HfO_2, annealed HfO_2, HZH, and ZHZ

Figure S8. Arrhenius plot of flatband voltage shift (ΔV_{FB}) measured at a stress time of 100 s under a negative bias stress of –2 V

Figure S9. Flatband voltage shift (ΔV_{FB}) measured at stress times of 1 ms, 10 ms, 100 ms, 1 s, and 10 s under negative bias stress ranging from 0 V to –3 V

Figure S10. Flatband voltage shift under NBS stress at RT and 85 °C for different dipole locations and annealing temperatures

Note S1: Fabrication of MOS capacitors

Note S2: Device measurement

Note S3: V_{FB} extraction method

Note S4: Hauser's model for ultrathin dielectric CV measurement

Note S5: Trap-induced flatband shift under negative bias stress

Note S6: Dipole effect in high-κ gate stacks

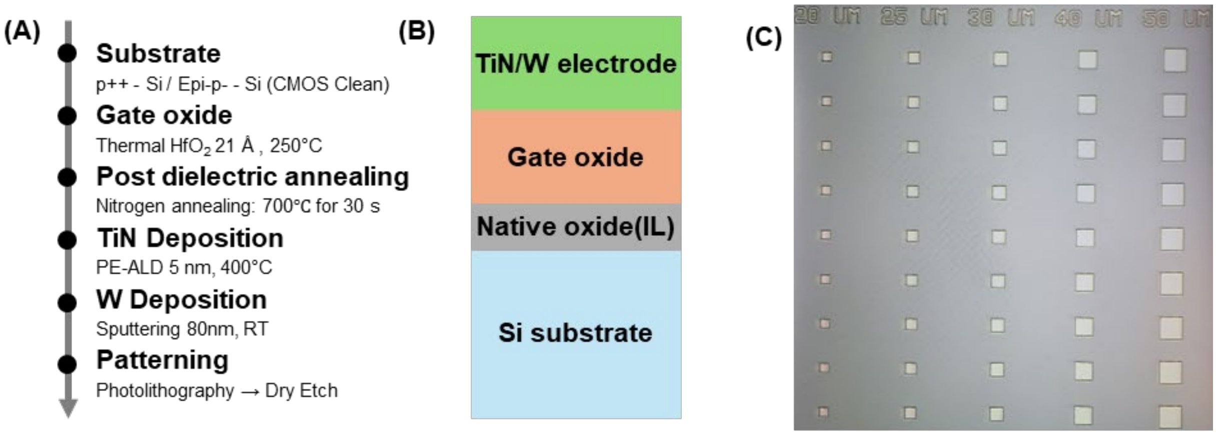

Figure S1. 21 Å Gate-Oxide MOSCAP: Fabrication Steps, Stack Schematic, and Chip Layout (a) Process flow for the fabricated MOS capacitor featuring a 21 Å gate-oxide layer. (b) Schematic cross-section of the vertical MOSCAP stack. (c) Optical-microscope layout image of the MOS capacitor.

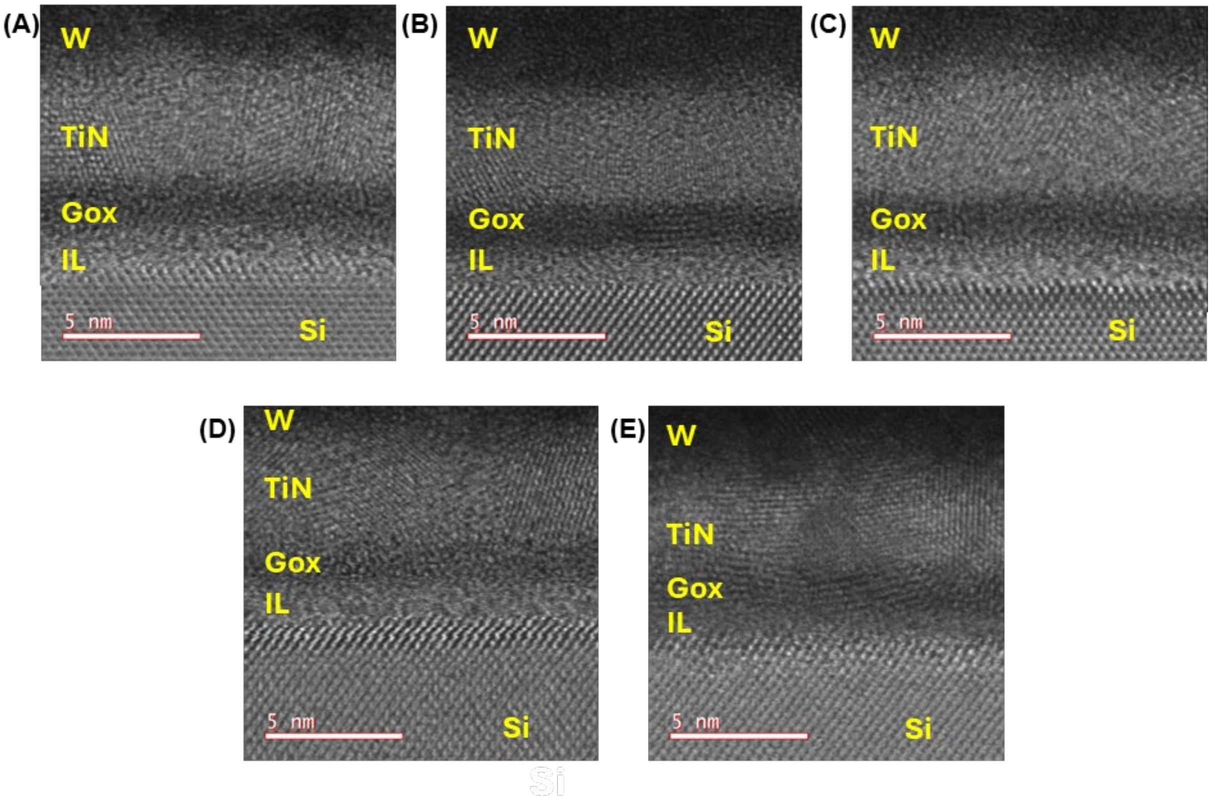

Figure S2. Cross-Section TEM Images of Multiple Gate-Stack Architectures (A) Conventional 21 Å HfO_2 without post-deposition annealing (B) Conventional 21 Å HfO_2 after post-deposition annealing (C) 18 Å HfO_2 with a 3 Å Al_2O_3 bottom dipole (D) 7 Å HfO_2–7 Å ZrO_2–7 Å HfO_2 (HZH) (E) 4 Å HfO_2–10 Å ZrO_2–4 Å HfO_2 (HZH) with a 3 Å Al_2O_3 bottom dipole.

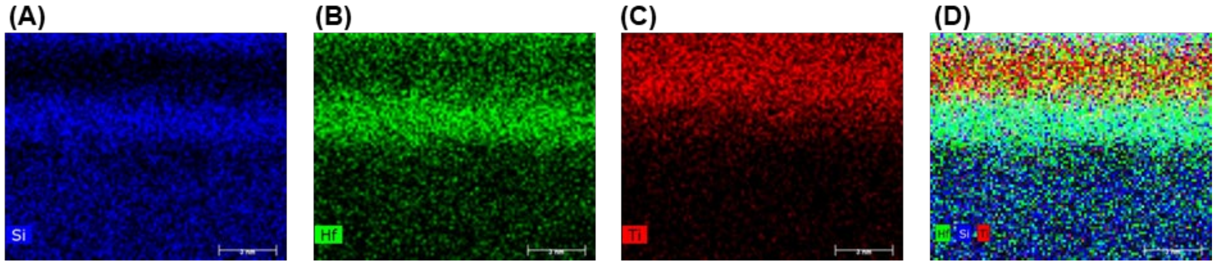

Figure S3. EDS Mapping of 21 Å HfO_2 Gate Stack (A) Si map (B) Hf map (C) Ti map (D) composite overlay of all three. Because the oxygen K-edge overlaps with the Ti Lα-edge, its elemental map appears delocalized. Likewise, the Si Kα-edge overlaps with the Hf M-edge.

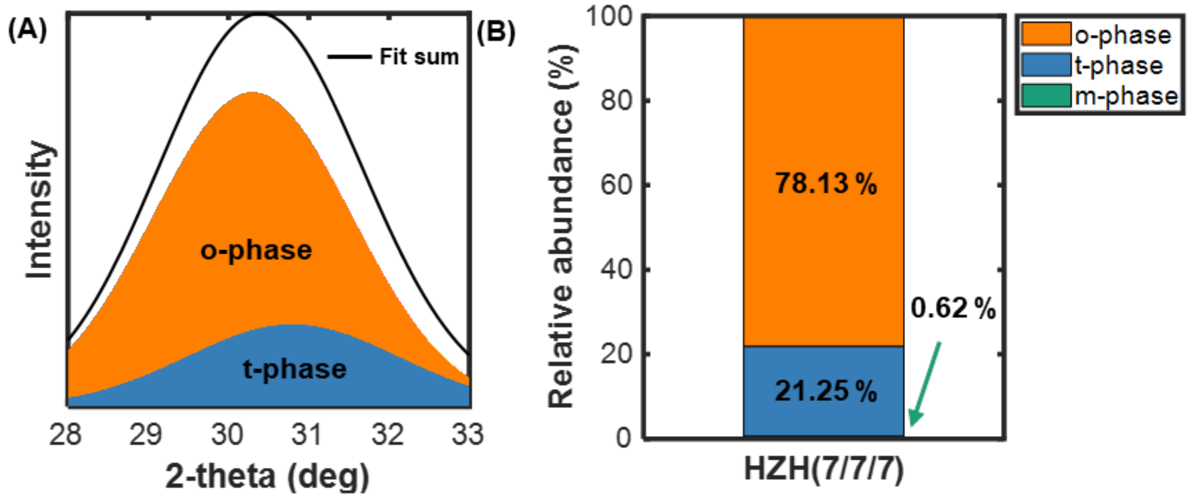

Figure S4. XRD analysis of HfO_2-ZrO_2-HfO_2 superlattice gate oxide (A) XRD spectrum of the HZH(7/7/7) film showing peaks near 30.5°. (B) Relative phase abundance indicating dominant o-phase in the HZH film.

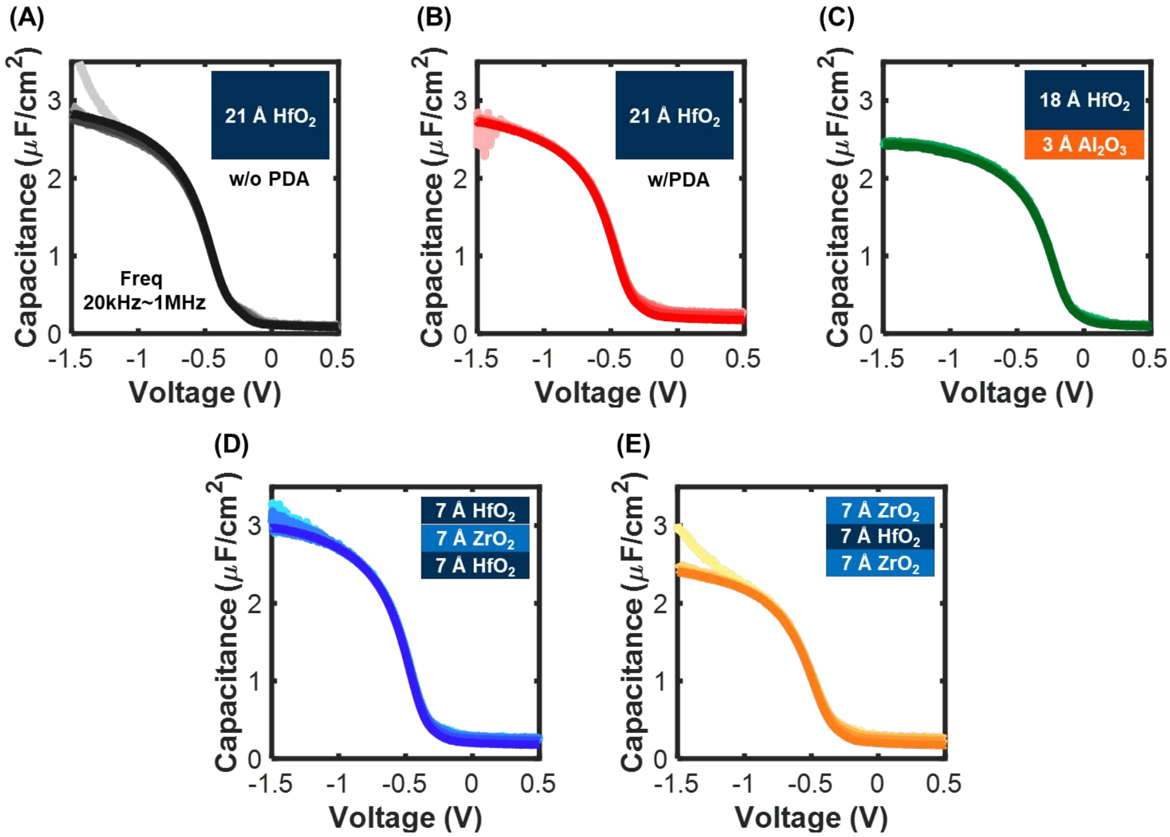

Figure S5. Frequency-dependent capacitance–voltage characteristics of various gate stacks (A)–(E) C–V measurements (20 kHz to 1 MHz) for gate stacks consisting of (A) 21 Å HfO_2 without PDA, (B) 21 Å HfO_2 with PDA, (C) HA, (D) HZH(7/7/7), and (E) ZHZ(7/7/7).

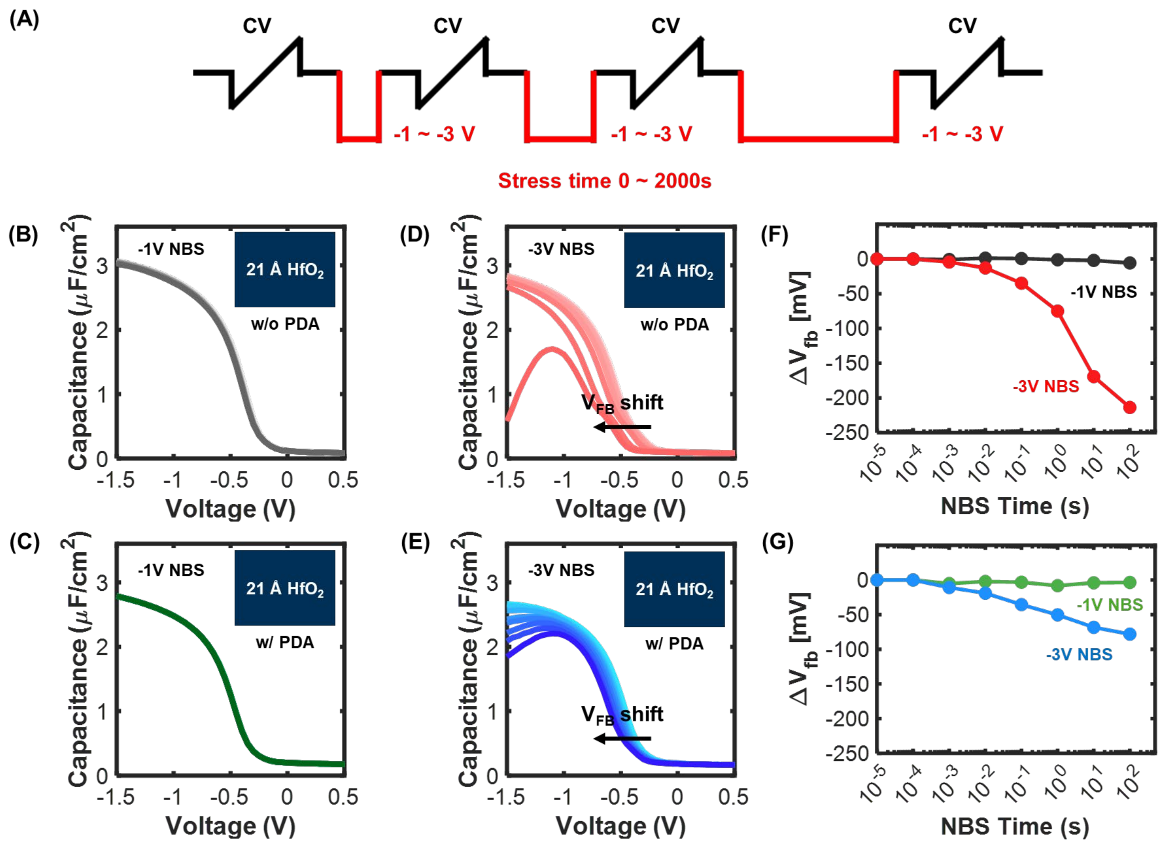

Figure S6. NBS stress comparison of gate stacks under different stress biases

(A) Schematic of the negative bias stress (NBS) test sequence. (B)–(C) Capacitance–voltage (C–V) measurements after –1 V NBS stress applied for 0 to 2000 s. (D)–(E) Capacitance–voltage (C–V) measurements after –3 V NBS stress applied for 0 to 2000 s. (F) Flatband voltage shift (ΔV_{FB}) over time under –1 V and –3 V NBS for the sample without PDA. (G) Flatband voltage shift (ΔV_{FB}) over time under –1 V and –3 V NBS for the sample with PDA.

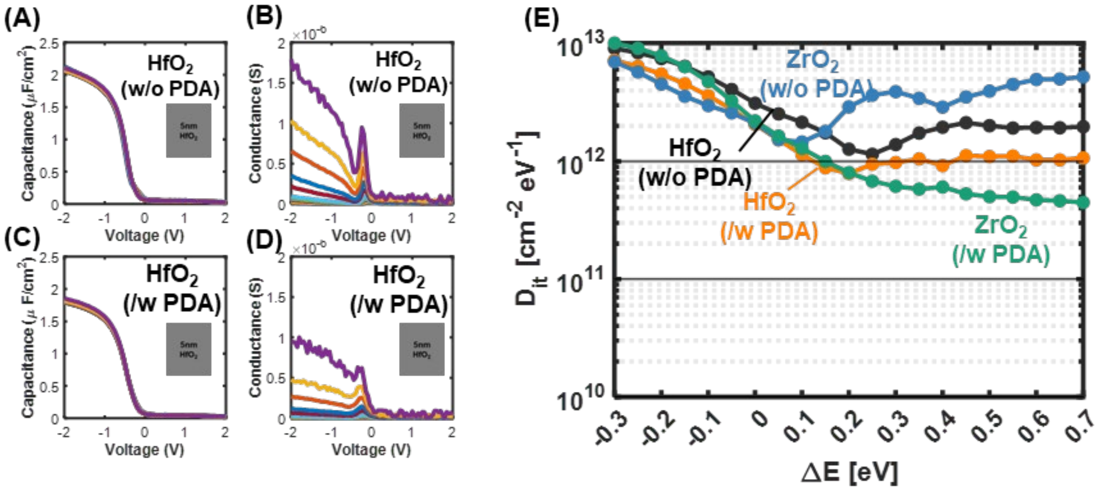

Figure S7. Interface trap density (D_{it}) comparison for 5 nm-thick HfO_2 and ZrO_2-based gate stacks with and without post-deposition annealing. (A) Capacitance–voltage (C–V) characteristics measured from 10 kHz to 1 MHz for unannealed HfO_2 (B) Conductance–voltage (G–V) characteristics for unannealed HfO_2 over the same frequency range (C) C–V measurement for annealed HfO_2, (D) G–V measurement for annealed HfO_2, and (E) Extracted D_{it} as a function of energy for various gate stack configurations using the conductance method.

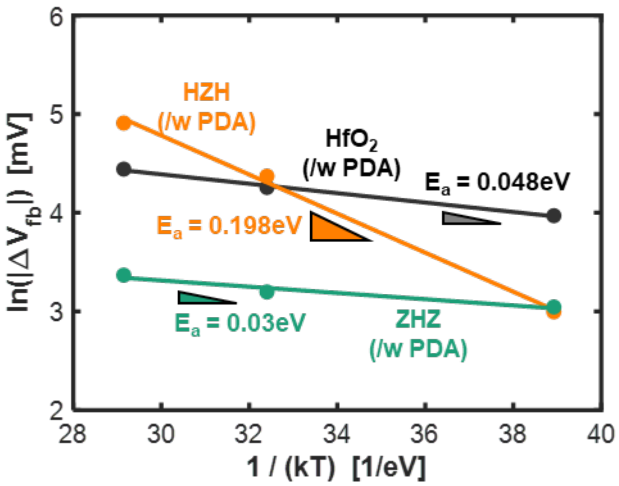

Figure S8. Arrhenius plot of flatband voltage shift (ΔV_{FB}) measured at a stress time of 100 s under a negative bias stress of –2 V. Each data point represents measurements at three temperatures (RT = 25 °C, 85 °C, and 125 °C). The activation energy (Ea) for each sample was extracted from the linear extrapolation of these three ΔV_{FB} data points plotted as $\ln|\Delta V_{FB}|$ versus 1/kT.

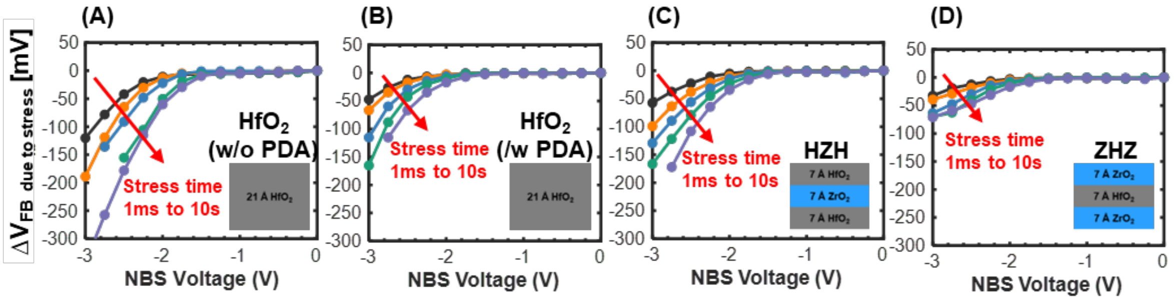

Figure S9. Flatband voltage shift (ΔV_{FB}) measured at stress times of 1 ms, 10 ms, 100 ms, 1 s, and 10 s under negative bias stress ranging from 0 V to –3 V. (A) conventional HfO_2 without post-deposition annealing (PDA), (B) conventional HfO_2 with PDA, (C) HZH superlattice with PDA, and (D) ZHZ superlattice with PDA

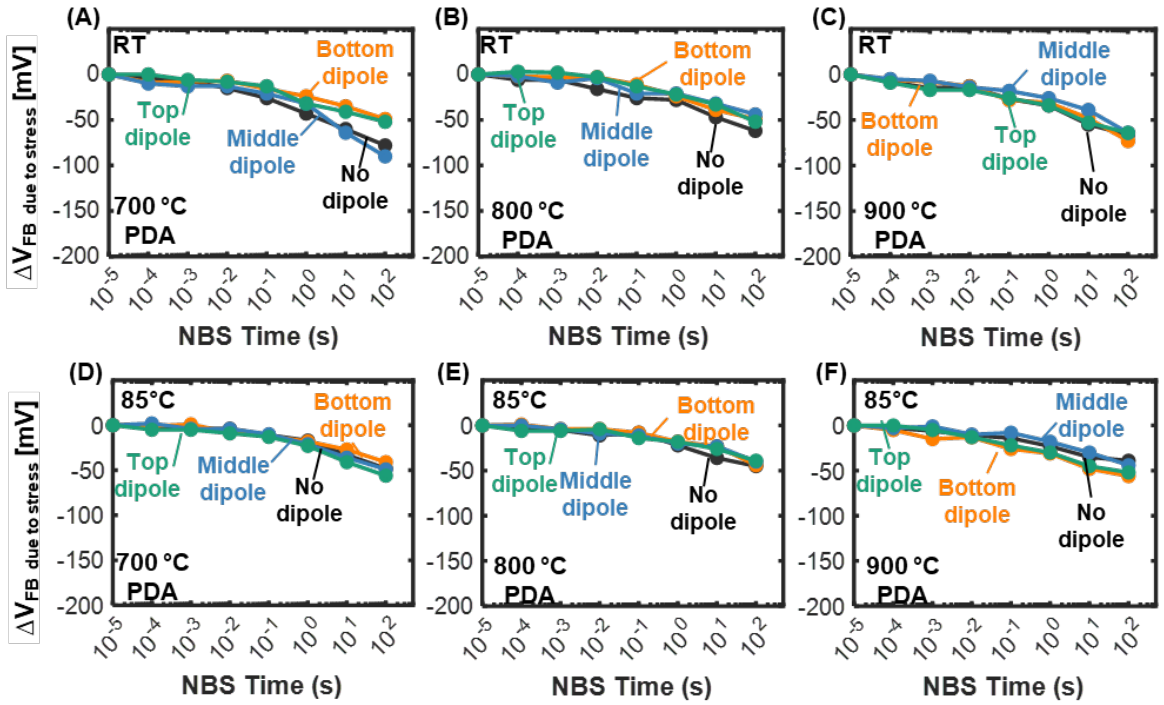

Figure S10. Flatband voltage shift under NBS stress at RT and 85 °C for different dipole locations and annealing temperatures. (A)–(C) ΔV_{FB} evolution under –2 V negative bias stress at room temperature as a function of time for samples annealed at (A) 700 °C, (B) 800 °C, and (C) 900 °C, comparing gate stacks with top, middle, bottom, and no dipole configurations. (D)–(F) ΔV_{FB} evolution under –2 V negative bias stress at 85°C as a function of time for samples annealed at (D) 700 °C, (E) 800 °C, and (F) 900 °C, comparing gate stacks with top, middle, bottom, and no dipole configurations.

Note S1: Fabrication of MOS capacitors

MOS capacitors with various gate stacks composed of HfO_2, ZrO_2, and Al_2O_3 were fabricated using the process flow shown in Figure S1. All devices were fabricated on a p++ silicon wafer with an epitaxially grown p-- silicon layer. the wafers underwent cleaning with Piranha solution (9:1 mixture of H_2SO_4 and H_2O_2) at 120 °C for 10 minutes, followed by a 30-second treatment with dilute HF (20:1) at room temperature. A 10-minute rinse in SC1 solution (5:1:1 mixture of H_2O, NH_4OH, and H_2O_2) at 75 °C was then performed to remove residual contaminants and oxide from the substrate, utilizing an RCA CMOS cleaning station. The HfO_2, ZrO_2, and Al_2O_3 layers were deposited in-situ by thermal atomic layer deposition (thermal-ALD) at 293 °C. The precursors for hafnium, zirconium, and aluminum were tetrakis(dimethylamido)hafnium (TDMaHf), tetrakis(dimethylamido)zirconium (TDMaZr), and trimethylaluminum (TMA), respectively, with H_2O as the oxygen source. Following deposition, the devices underwent post-dielectric annealing (PDA) at 700 °C for 30 seconds in a nitrogen atmosphere. The top electrode was fabricated using a combination of TiN and W. TiN was deposited at 448 °C by plasma enhanced atomic layer deposition (PE-ALD) using TiCl4 precursor and NH_3 gas, followed by sputtering of tungsten onto the structure. For photo patterning, SC1813 photoresist was spin-coated onto the wafers, followed by exposure using a Heidelberg MLA150. The pattern was then developed by immersing the photoresist in MF-319 for 30 seconds. After development, the pattern was etched using reactive ion etching (RIE) in an SF_6/O_2 gas atmosphere, effectively etching the tungsten (W) and titanium nitride (TiN) layers.

Note S2: Device measurement

The electrical characteristics of all devices were evaluated using a Keysight B1500A semiconductor device analyzer. Capacitors with a size of 20 × 20 μm^2 were measured, and capacitance-voltage (C-V) measurements were performed at a frequency of 1MHz. The waveform sequences used for negative bias stress (NBS) measurements are shown in Figure S6(A). A negative bias of -2 V was applied and increased stepwise from 0 seconds to 2000 seconds, with a 1 second refresh interval between each stress and subsequent measurement. NBS measurements were performed using the measurement-stress-measurement (MSM) method, where C-V measurements were taken both before and after applying stress. For NBS testing, measurements were carried out at both room temperature and high temperature (125°C), with the chuck heated to the desired temperature to study NBS behavior under different thermal conditions. Since the thickness of the interlayer (SiO_2) was confirmed with a transmission electron microscopy (TEM) image to be approximately 10 Å, the electric field (E-field) across the dielectric is estimated to be around 6 MV/cm.

Note S3: V_{FB} extraction method

The capacitance values obtained from the C-V measurements were used to calculate the flatband capacitance (C_{FB}), and the corresponding voltage was defined as the flat-band voltage (V_{FB}). The formula for calculating the C_{FB} is given in (1).

$$\frac{1}{C_{FB}} = \frac{1}{C_{ox}} + \frac{1}{C_{debye}} \quad (1)$$

Here, oxide capacitance (C_{ox}) was determined using the maximum capacitance value (C_{max}), as the device is in accumulation mode and saturated, allowing Cox to be reliably extracted from C_{max}. The Debye capacitance (C_{debye}) was calculated as follows (2).

$$\frac{1}{C_{debye}} = \frac{\varepsilon_0 \varepsilon_{Si}}{L_D} \quad (2)$$

The permittivity of vacuum (ε_0) was taken as 8.854 × 10^{-14} F/cm, and the relative permittivity of silicon (ε_{si}) was set to 11.9. The Debye length (L_D) was determined using the following (3).

$$L_D = \sqrt{\frac{k_B T \varepsilon_{Si}}{N_A q^2}} \quad (3)$$

In this calculation, N_A was derived from the resistivity of the wafer and assumed to be 9.7×10^{15} cm^{-3}. The Boltzmann constant (k_B) was set to 1.38×10^{-23} J/K, the temperature (T) to 300 K, and the electron charge (q) to 1.6×10^{-19} C. The voltage corresponding to the calculated C_{FB} was then defined as V_{FB}. Since gate leakage current density (J_g) increases with applied voltage, the J_g value in this study was measured at V_{FB} - 1 V to standardize the results. Given that the studied film thickness is only 21 Å, which is extremely thin, quantum mechanical effects make it challenging to directly represent the equivalent oxide thickness (EOT) using capacitance

equivalent thickness (CET). Therefore, the Hauser model[1] was employed to convert the CET values to EOT.

Note S4: Hauser's model for ultrathin dielectric CV measurement

When the gate dielectric thickness $t_{physical}$ approaches the sub-2 nm regime, classical capacitance models ($C = \varepsilon_0 \kappa / t_{ox}$) fail to capture two qualitatively new effects: first, in strong inversion the carriers occupy quantized subbands whose charge centroid lies a finite distance $T_{inversion}$ (typically 1–3 nm) below the Si surface, creating a "dead-layer" of charge that effectively appears as an additional series capacitance on the semiconductor side; second, the carrier wave-function extends partially into the oxide, further reducing the net capacitance relative to the classical prediction.

Hauser's model corrects for these quantum mechanical effects by introducing an additional series capacitance, $C_{inversion}$, that represents the inversion-layer response. The total gate capacitance per unit area, C_{total}, then becomes:

$$\frac{1}{C_{total}} = \frac{1}{C_{ox}} + \frac{1}{C_{inversion}} \tag{1}$$

where

$$C_{ox} = \frac{\varepsilon_0 \kappa_{SiO_2}}{t_{physical}} \quad \text{and} \quad C_{inversion} = \frac{\varepsilon_0 \kappa_{Si}}{T_{inversion}} \tag{2}$$

Here κ_{ox} and $\kappa_{Si} \approx 11.7$ are the dielectric constants of the gate oxide and silicon, respectively. Equation (1) yields an effective electrical thickness

$$t_{EOT} = \frac{\varepsilon_0 \kappa_{SiO_2}}{C_{total}} \approx t_{physical} + \frac{\kappa_{SiO_2}}{\kappa_{ox}} T_{inversion} \tag{3}$$

where $\kappa_{SiO_2} = 3.9$. The second term,

$$\Delta t_q = \frac{\kappa_{SiO_2}}{\kappa_{ox}} T_{inversion} \tag{4}$$

is the quantum correction that accounts for both carrier centroid displacement and wave-function tailing.[2, 3]

In practice, Hauser's model is integrated into a C–V fitting routine: one varied the physical oxide thickness $t_{physical}$, the inversion centroid position $T_{inversion}$, and the flat-band voltage V_{FB} (including any interfacial dipole contribution), until the simulated high-frequency C–V curve matches the measurement. The resulting EOT thus self-consistently includes classical dielectric scaling, quantum confinement, and any fixed interfacial dipoles, providing an accurate extraction of the sub-1 nm EOT from experimental data.

Note S5: Trap-induced flatband shift under negative bias stress

In ultrathin high-κ/SiO_2 gate stacks subjected to negative gate bias, two distinct trap populations contribute to the observed flat-band voltage shift, ΔV_{FB}: fast interface states and slow bulk (or border-oxide) traps.[4-7] Interface states—predominantly silicon dangling-bond (Pb) centers—form when Si–H or Si–Si bonds at the SiO_2/Si interface are broken by hole capture. Because these traps lie within a few angstroms of the channel, they exchange charge on microsecond to millisecond time scales.[8] Experimentally, the interface-trap sheet density, ΔN_{it} (t), follows a sublinear power law with stress time t:

$$\Delta N_{it}(t) = At^{n},\ n \approx 0.1 - 0.3 \tag{1}$$

where A is bias- and temperature-dependent. The positive charge generated at the interface, $Q_{it} = \mathrm{q}\Delta N_{it}(t)$, produces an immediate shift in flat-band voltage

$$\Delta V_{FB,it}(t) = -\frac{Q_{it}}{C_{ox}} = -\frac{\mathrm{q}\Delta N_{it}(t)}{C_{ox}} \tag{2}$$

Because interface traps lie energetically near the silicon valence-band edge, they form readily under negative bias and dominate the early-time ΔV_{FB} response.

Simultaneously, holes tunnel into deeper defect levels in the high-κ layer or the interfacial SiO_2, becoming trapped at oxygen vacancies, hydrogen-related complexes, or other bulk defects. These bulk or border-oxide traps are located several nanometers from the channel, incur significant lattice relaxation, and thus exchange charge on time scales ranging from seconds to hours. Their contribution to flat-band shift is often described by a logarithmic dependence:

$$\Delta N_{ot}(t) \propto \ln{(t/t_0)},\ \Delta V_{FB,ot}(t) = -\frac{\mathrm{q}\Delta N_{ot}(t)}{C_{ox}} \tag{3}$$

where t_0 is a reference time. Consequently, $\Delta V_{FB}(t)$ exhibits an initial rapid rise (interface-trap–dominated) followed by a slower, near-logarithmic tail (oxide-trap–dominated). Fitting $\Delta V_{FB}(t)$ with the sum of (1) and (2) allows separation of the fast and slow trap densities and kinetics.[6, 7, 9, 10]

The temperature dependence of these processes further distinguishes them: interface-state generation requires overcoming bond-breaking activation energies (E_A~0.1–0.3eV) and thus accelerates markedly with temperature, whereas hole trapping in pre-existing oxide defects shows much weaker thermal activation. As a result, elevated stress temperatures amplify the early interface component of ΔV_{FB}, while the long-time oxide-trap contribution remains relatively unchanged. Understanding the interplay of these two trapping mechanisms is essential for accurate reliability projections of sub-nanometer EOT gate dielectrics under negative bias stress.

Note S6: Dipole effect in high-κ gate stacks

In high-κ/SiO_2 gate stacks, engineered interfacial dipoles arise from the intrinsic mismatch in oxygen areal density and cation electronegativity at oxide–oxide junctions.[11, 12] During post-deposition annealing, oxygen ions diffuse from the higher-density layer (e.g., SiO_2) into the adjacent lower-density high-κ dielectric (e.g., HfO_2), leaving behind positively charged vacancies; concurrently, the more electronegative cations at the heterojunction attract electron density, creating an atomic-scale separation of charge.[13, 14] This fixed dipole moment per unit area, P, produces a localized potential step

$$\Delta\phi = -\frac{P}{\varepsilon_0} \quad (1)$$

and thus a rigid flat-band voltage shift

$$\Delta V_{FB} = -\frac{P}{\varepsilon_0} = -\frac{\mu}{\varepsilon_0 A} \quad (2)$$

where μ is the total dipole moment on area A and ε_0 the vacuum permittivity. Equivalently, treating the dipole as an equivalent sheet charge ΔQ=P/d (with effective separation d) yields[15]

$$\Delta V_{FB} = -\frac{\Delta Q}{C_{ox}} = -\frac{P/d}{\varepsilon_0 \kappa_{ox}/t_{ox}} \quad (3)$$

demonstrating that ΔV_{FB} is largely independent of the bulk oxide thickness t_{ox}. Inserting an ultrathin (3 Å) Al_2O_3 interlayer at the SiO_2/HfO_2 interface creates two high-polarity junctions (SiO_2/Al_2O_3 and Al_2O_3/HfO_2), each contributing to P. Empirical C–V measurements confirm that, following a 700 °C N_2 anneal, these Al_2O_3-enabled dipoles induce flat-band shifts exceeding 200 mV without degrading leakage current. Thus, interfacial dipole engineering via controlled insertion of high-oxygen-density

layers offers a powerful, thickness-agnostic mechanism for precise V_{th} tuning in sub-nanometer EOT gate stacks, combining robust thermal process compatibility with minimal EOT penalty and preserved reliability.

Supplementary References:

(1) Hauser, J. R.; Ahmed, K. Characterization of ultra-thin oxides using electrical C-V and I-V measurements. In The 1998 international conference on characterization and metrology for ULSI technology, 1998.
(2) Anwar, S. R. M.; Vandenberghe, W. G.; Bersuker, G.; Veksler, D.; Verzellesi, G.; Morassi, L.; Galatage, R. V.; Jha, S.; Buie, C.; Barton, A. T.; et al. Comprehensive Capacitance–Voltage Simulation and Extraction Tool Including Quantum Effects for High-k on Six/Ge1-x and InxGa1-xAs: Part I—Model Description and Validation. *IEEE Transactions on Electron Devices* **2017**, *64* (9), 3786-3793. DOI: 10.1109/ted.2017.2725645.
(3) Hu, C. Modern semiconductor devices for integrated circuits. *(No Title)* **2010**.
(4) Jeppson, K. O.; Svensson, C. M. Negative bias stress of MOS devices at high electric fields and degradation of MNOS devices. *Journal of Applied Physics* **1977**, *48* (5), 2004-2014. DOI: 10.1063/1.323909.
(5) Stathis, J. H.; Zafar, S. The negative bias temperature instability in MOS devices: A review. *Microelectronics Reliability* **2006**, *46* (2-4), 270-286. DOI: 10.1016/j.microrel.2005.08.001.
(6) Rahim, N.; Misra, D. Role of hydrogen in Ge⁄HfO2⁄Al gate stacks subjected to negative bias temperature instability. *Applied Physics Letters* **2008**, *92* (2). DOI: 10.1063/1.2827567.
(7) Gao, Y.; Ang, D. S.; Gu, C. J. On the Evolution of Switching Oxide Traps in the HfO2/TiN Gate Stack Subjected to Positive- and Negative-Bias Temperature Stressing. *ECS Transactions* **2013**, *53* (3), 205-220. DOI: 10.1149/05303.0205ecst.
(8) Zhu, B.; Suehle, J. S.; Voge, E.; Bernstein, J. The contribution of HfO/sub 2/bulk oxide traps to dynamic NBTI in pMOSFETs. In *2005 IEEE International Reliability Physics Symposium, 2005. Proceedings. 43rd Annual.*, 2005; IEEE: pp 533-537.
(9) Liu, Y.-Y.; Liu, F.; Wang, R.; Luo, J.-W.; Jiang, X.; Huang, R.; Li, S.-S.; Wang, L.-W. Characterizing the Charge Trapping across Crystalline and Amorphous Si/SiO2/HfO2 Stacks from First-Principle Calculations. *Physical Review Applied* **2019**, *12* (6). DOI: 10.1103/PhysRevApplied.12.064012.
(10) Stampfer, B. Advanced electrical characterization of charge trapping in MOS transistors. Technische Universität Wien, 2020.
(11) Lv, Y.-D.; Shen, L.; Li, Y.-C.; Shi, C.-Y.; Huang, Z.-Y.; Yu, X.; Zhu, X.-N.; Lu, H.-L.; Yu, S.; Zhang, D. W. Atomic-Layer-Deposited Al2O3 Layer Inserted in SiO2/HfO2 Gate-Stack-Induced Positive Flat-Band Shift with Dual Interface Dipoles for Advanced Logic Device. *ACS Applied Nano Materials* **2024**, *7* (24), 28496-28503. DOI: 10.1021/acsanm.4c05649.
(12) Zhang, Y.; Choi, M.; Wang, Z.; Choi, C. Dipole formation to modulate flatband voltage using ALD Al2O3 and La2O3 at the interface between HfO2 and Si or Ge substrates. *Applied Surface Science* **2023**, *609*. DOI: 10.1016/j.apsusc.2022.155295.
(13) Arimura, H.; Ragnarsson, L. A.; Oniki, Y.; Franco, J.; Vandooren, A.; Brus, S.; Leonhardt, A.; Sippola, P.; Ivanova, T.; Verni, G. A.; et al. Dipole-First Gate Stack as a Scalable and Thermal Budget Flexible Multi-Vt Solution for Nanosheet/CFET Devices. In 2021 IEEE International Electron Devices Meeting (IEDM), 2021.
(14) Zheng, D.; Chung, W.; Chen, Z.; Si, M.; Wilk, C.; Ye, P. D. Controlling Threshold Voltage of CMOS SOI Nanowire FETs With Sub-1 nm Dipole Layers Formed by Atomic Layer Deposition. *IEEE Transactions on Electron Devices* **2022**, *69* (2), 851-856. DOI: 10.1109/ted.2021.3136493.

(15) Kita, K.; Kamata, H.; Fei, J. Interface Dipole Layers between Two Dielectrics: Considerations on Physical Origins and Opportunities to Control Their Formation. In *Int. Conf. Solid State Devices & Materials*, 2017; pp 499-500.
